# A tutorial on the physics of light and image shading

**DOI:** 10.1177/20416695241279929

**Published:** 2024-09-30

**Authors:** James T. Todd

**Affiliations:** Department of Psychology, 2647 Ohio State University, Columbus, OH, USA

**Keywords:** optics, image shading, shape perception, material perception

## Abstract

This paper provides an overview of the many different ways that light interacts with surfaces in the natural environment to provide useful information for visual perception. It begins with a discussion of how the concept of light has evolved over the course of human history. It then considers a wide variety of optical phenomena including Lambert's laws of illumination, the effects of microscopic surface structure on patterns of reflection, the bidirectional reflectance distribution function, the refraction of transmitted light, chromatic dispersion, thin film interference, sub-surface scattering, the Fresnel effects, indirect illumination from multiple reflections, caustics, and the structure of the light field. The primary goal of this discussion is to provide the necessary background information to help students and young researchers more easily understand the scientific literature on the perception of 3D shape and material properties from patterns of image shading.

The use of computer graphics has a long history in the study of visual perception. It began in the 1950s with the pioneering research of Gunnar Johansson, who used oscilloscope technology to investigate observers’ perceptions of complex patterns of motion. In the 1980s, a new technology called raster graphics became available that allowed researchers to manipulate the color and gray scale of computer graphics displays. This ushered in the first research on the visual perception of 3D shape and material properties from gradients of image shading (Bülthoff & Mallot, 1988; Mingolla & Todd, 1986; Todd & Mingolla, 1983). Although those early investigations were quite primitive, the hardware and software for simulating patterns of light within complex scenes have improved dramatically over the past four decades, and it is now possible to study a wide variety of perceptual phenomena that would have been difficult to achieve until quite recently.

One impediment to the development of this research is that sophisticated analyses of ecological optics are not generally taught in graduate training programs in perceptual psychology. Most students who are enrolled in those programs have little or no knowledge of basic physics or computer graphics, and those with an interest in these topics are left to fend for themselves. As a result, much of what they learn is based on trial and error while playing around with 3D rendering software. The present article is intended to provide a useful resource for students and young researchers in visual perception by providing an overview of the basic physical principles that govern how the pattern of light at a point of observation is structured by visible surfaces in the natural environment.

There are many different software packages for creating shaded images of 3D scenes, and there are large variations among them in terms of speed and accuracy. Some popular renderers used for commercial applications include Arnold, Corona, Cycles, Maxwell, Mitsuba, Octane, Redshift, and V-Ray. Most of the images presented in this article were created using the Maxwell renderer because of its physically accurate lighting and materials. There is, however, a cost for that accuracy. Some of these images required several hours of processing on a dedicated computer cluster. Most of the effects described in this paper can be achieved with any of the renderers mentioned above, though there are a few effects that can only be approximated or are not implemented on some renderers.

The tutorial is organized into eight sections. Section 1 provides an overview of how the concept of light has evolved over the course of human history. Section 2 describes Lambert's laws of illumination. Section 3 discusses the reflection of light, including bidirectional reflectance distribution functions, and the effects of wavelength that are responsible for color perception. Section 4 considers a variety of factors that influence the transmission of light, including the effects of refraction, chromatic dispersion, thin film interference, and subsurface scattering. Section 5 discusses the Fresnel effects that govern the relative proportion of reflection and transmission as a function of the incident angle of illumination. Section 6 considers how patterns of image shading are influenced by indirect illumination from the reflections of neighboring surfaces. Section 7 introduces the concept of the light field and how it interacts with different types of surface materials. Finally, Section 8 considers some practical implications of this discussion for psychological research.

## Section 1: The History of Light as a Scientific Concept

### Geometrical Optics

The phenomenon of vision has fascinated scientists and philosophers since the dawn of civilization, but our conceptual understanding about the concept of light has evolved slowly over the past three millennia. For example, the ancient Egyptians believed that light was produced by the eye of the sun god Ra. Daylight occurs when his eye is open, and night falls when his eye is closed. In the 5th century BC, Plato and his followers argued that light consists of rays or particles emitted from the eyes. When these rays strike an object, it is like reaching out to touch it at a distance, and that is what allows us to perceive an object's size, shape, or color. This emission theory of light was the dominant view for almost 1000 years until it was eventually disproved by the great Arabic scholar Alhazan. He showed that light is emitted from luminous sources such as lanterns or the sun rather than the eye.

**Figure 1.**
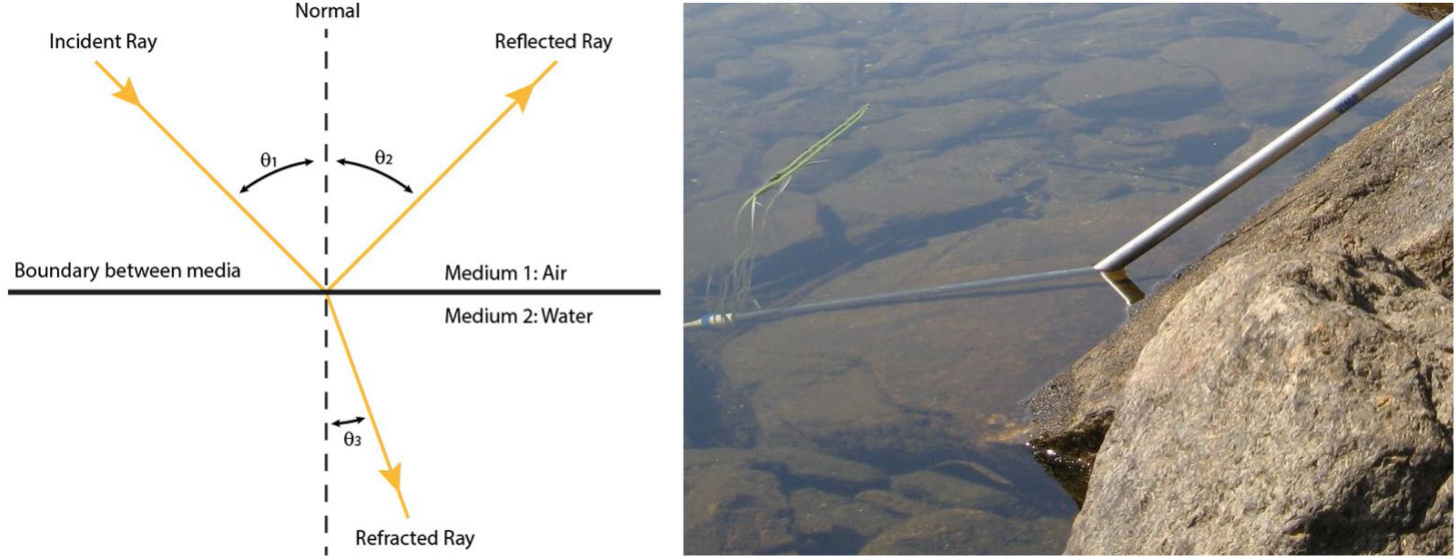
Left: A diagram that shows a light ray with an incident angle (*θ*_1_) relative to a pool of water, and its angles of reflection (*θ*_2_) and refraction (*θ*_3_). Right: A bent pole illusion caused by the refraction of light as it passes from air to water. This photograph was taken by Delamaran and is licensed under creative commons CC0.

Despite their misconceptions about the nature of light, the ancient Greeks were able to accurately describe many of its properties based solely on the idea that the emitted rays move in straight lines (Zubairy, 2016). This general approach is often referred to as geometrical optics. The Greeks were obviously aware of mirror reflections as is evident in the myth of Narcissus who fell in love with his own reflection. Hero of Alexandria (10–70) was able to explain reflection off smooth surfaces using a principal of least distance to show that the angle of incidence is equal to the angle of reflection (see the left panel of Figure 1). A similar idea is implicit in the earlier work of Diocles (240–180 BC). He wrote a work called “Burning mirrors” that showed how a parabolic mirror (or lens) can be used to focus parallel light rays onto a single point, which can be exploited to create fire. Little remains of this original manuscript except for a few fragments that were copied in the 15th century (Toomer, 1976). This work was particularly influential on Arabic mathematicians such as Alhazan.

The ancient Greeks also knew about the refraction of light when it passes from one medium to another (see the right panel of Figure 1). Claudius Ptolemy (90–168) studied this phenomenon in a careful series of experiments and he concluded that the angle of refraction is a constant ratio of the angle of incidence based on the properties of the two media. This is a small angle approximation of the modern law of refraction proposed by Willebrord Snellius (1580–1626). However, that law was actually discovered 600 years earlier by the Arabic mathematician Ibn Sahl (Zubairy, 2016).

### Physical Optics

**Figure 2.**
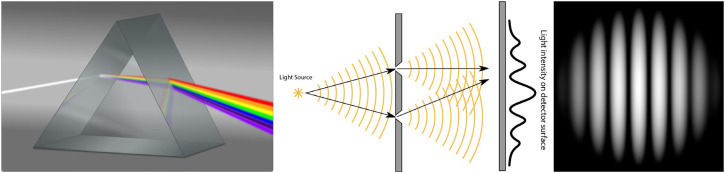
Left: A diagram of light refraction through a wedge prism by Goran-tek-en, licensed under CC-SA-4.0. Note that violet light with a small wavelength bends more than red light with a longer wavelength. Middle: A diagram of the two slit experiment performed by Thomas Young. Right: A simulated diffraction grating by Timm Weitkamp licensed under CC-BY-3.0.

Although Plato's emission theory of light was eventually abandoned, the idea that light is composed of particles persisted until the 19th century. This is referred to as the corpuscular theory of light. The first challenge to that idea was discovered by Francesco Grimaldi (1618–1663). He showed that when light passes through a hole, it does not follow a rectilinear path as would be expected based on the corpuscular theory. Rather, it fans out from the hole in the shape of a cone, just like the diffraction of waves in air or water. Another puzzling phenomenon studied by Sir Isaac Newton (1642–1727) is the dispersive refraction of light through prisms. He showed that when light from the sun passes through a prism it fans out into a spectrum of colors (see the left panel of Figure 2), but this does not occur if light of a single color is passed through the prism. He also showed that when multiple colors of light are combined, they appear white, thus proving that white light is composed of multiple colors. Despite these observations, Newton was reluctant to abandon the corpuscular theory of light, and to accept the idea that light might be composed of waves.

More compelling evidence for the wave theory of light was provided by Thomas Young (1773–1829). He performed an experiment in which a beam of homogeneous light was projected onto a screen with two small slits that were spatially separated from one another (see the middle panel of Figure 2). When light fanned out from the slits, it created a pattern of interference called a diffraction grating as shown in the right panel of Figure 2. This was eventually accepted as definitive evidence that light is composed of waves. By measuring the distance between the peaks of the diffraction gratings for different colored lights, Young was also able to estimate the wave lengths of those colors. He showed that red light has the largest wavelength in the visible spectrum and that violet light has the smallest.

The description of light in terms of waves is now referred to as physical optics, as opposed to geometrical optics that describes the behavior of light in terms of particles or rays. Physicists in the 17th century were quite familiar with waves such as ocean waves or sound waves, but these examples all require a medium to travel through. What is the medium for light, given that it can propagate through solid matter such as glass, and also in the vacuum of empty space? Christian Huygens (1629–1695) proposed that the medium for light waves is a hypothetical substance called “ether” that exists everywhere in space.

**Figure 3.**
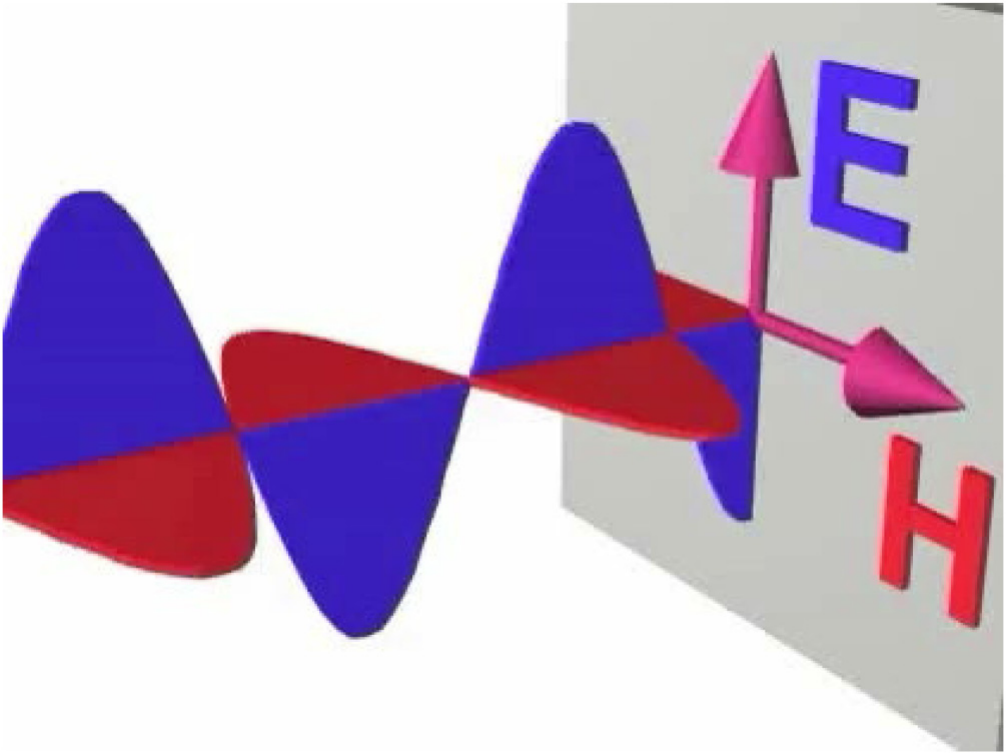
An animation of electromagnetic waves. Click image to start the animation.

James Clerk Maxwell (1831–1879) later showed that light waves involve transverse oscillations of electric field strength (E) and magnetic field strength (H), which are always oriented in orthogonal directions, as shown in Figure 3. The wavelengths of electromagnetic oscillations can vary from 10^−5^ nm for gamma rays to 10^3^ m for radio waves. Visible light consists of a small portion of that spectrum from 380–750 nm. Under natural lighting conditions, the absolute orientations of these oscillations vary randomly, and this is referred to as unpolarized light. However, it is possible to polarize light with filters so that all the waves oscillate in the same direction. Polarizing filters are typically used in sunglasses to reduce glare.

**Figure 4.**
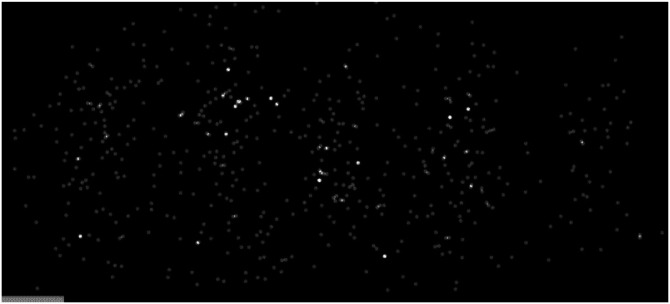
An animation from Bach et al. (2013) licensed under CC-BY-3.0. It shows the accumulation of photon detections over time in a double slit experiment. They initially appear to be randomly distributed, but they eventually form a pattern of light and dark bands. This suggests that the wave properties of light are a statistical distribution of individual photons. Click image to start the animation.

Despite the strong evidence that light is a wave, the corpuscular theory never really died. Albert Einstein (1905) proposed that light is made up of small packets of energy. He referred to these packets as light quanta, but they later became popularized using the term “photons” (Lewis, 1926). Consider a variant of the double slit experiment using an extremely weak light source with an extremely sensitive detector. When a photon collides with the detector, it is represented by a small dot. At first, the photons appear to be randomly distributed. However, as these collisions begin to accumulate, a pattern begins to emerge, in which they collectively form an alternating sequence of light and dark bands like those observed by Thomas Young. Figure 4 shows an animation sequence from such an experiment by Bach et al. (2013). This finding suggests that the wave properties of light are a statistical distribution of individual photons, which is sometimes referred to as the wave-particle duality.

### Ecological Optics

What does all of this have to do with visual perception? Although vision clearly involves sensitivity to light, the study of classical optics over the past three millennia provides little insight about how light informs a perceiving organism about the physical structure of its surrounding environment. The first serious attempt to address this issue was provided by James Gibson (1966). He described how the pattern of visual stimulation at a point of observation is structured by light that is scattered by surfaces in multiple directions. As the light bounces from one surface to another, it fills the volume of space with an infinitely dense network of intersecting rays at every point, and he referred to that structure as the “ambient optic array.” He also coined the term “ecological optics” for the scientific investigation of that structure.

**Figure 5.**
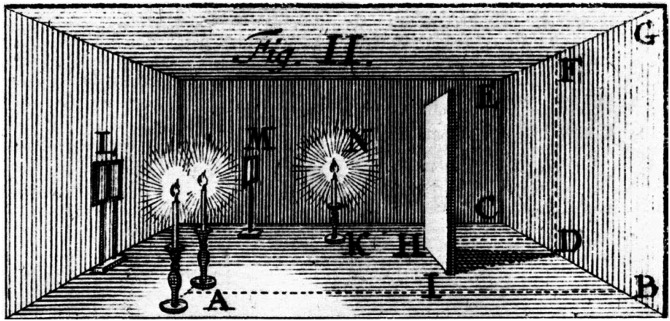
An illustration from Photometria (1760), which shows an optical configuration used by Lambert to study the effect of distance on illumination. Two regions of the back wall (CDEF on the left) and (DBFG on the right) are illuminated by candles at different distances and divided by a screen (HI). When viewed from location L, their relative brightness is compared based on visual observation. This image was scanned from the original book by Dufbug Deropa and is licensed under CC-BY-SA-3.0.

Gibson's proposal was a dramatic departure from traditional views about visual perception, but the roots of ecological optics were planted 200 years earlier by the Swiss physicist Johann Lambert. In his book Photometria (1760), Lambert reported the first systematic research on light that is scattered by microscopically rough surfaces. This work was remarkable in several important respects. At that time, there were no optical instruments to measure the intensity of light, so Lambert used the only reliable tool that was available—his own visual perception. For example, in one procedure shown in Figure 5, he used a screen to visually separate two regions of a blank wall. He placed a candle at a measured distance from one region, and he placed a second candle at a larger distance from the second region. When comparing the two regions, he could immediately see that the first one appeared brighter. To make them appear equal, he needed to place additional candles at the second location. Lambert performed 40 experiments using ingenious optical configurations. By systematically manipulating the physical parameters to make two adjacent regions appear equally bright, he was able to determine how those parameters influence a variety of optical properties such as illumination, reflectance, and transparency.

Lambert's research has had a huge impact on how we think about optics today, but it received relatively little attention during his lifetime. 18th century physicists were primarily concerned with the issue of whether light is composed of waves or particles, and Lambert's experiments were irrelevant to that issue. The scientific contributions of Photometria were not really appreciated until nearly a century after its publication, when his work provided some useful insights for the study of astronomy and the commerce of gas lighting. As the use of artificial lighting exploded in the early 20th century, illumination engineers used Lambert's results to calculate lighting in architectural designs, and his work also had an important influence on the development of computer graphics in the latter part of the 20th century.

## Section 2: Illumination

When light is emitted from a radiant source such as a candle it propagates outwards in all directions. Illumination (or illuminance) is defined as the magnitude of light energy that shines on a unit area of a surface. Lambert's experiments demonstrated that illumination of a surface is inversely proportional to the square of its distance from the light source $(I\propto 1/d^{2}$ ), and proportional to the cosine of the incident angle relative to the surface normal. Both of these effects can be explained theoretically by changes in the area of a surface over which a fixed quantity of light is distributed.

**Figure 6.**
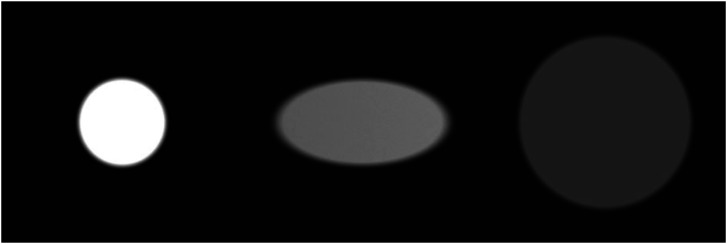
The optical projections of three identical lights at different distances and orientations relative to the display screen. Left: The projection of a cone of light with an incident angle of 0°. Middle: The projection of a light at the same distance with an incident angle of 60°. This doubles the area of the projection and reduces the illumination by half. Right: The projection of a light at twice the distance as the one on the left with an incident angle of 0°. This quadruples the area of the projection and reduces the illumination by 1/4.

To better appreciate Lambert's methods, it is useful to consider Figure 6, which shows three views of a fronto-parallel planar surface illuminated by identical light sources at different distances and orientations. The surface in the left panel is illuminated by a cone of light with an incident angle of 0° that creates a circular pattern of illumination. The surface in the middle panel is the same distance from the light source as the one on the left, but the incident angle of illumination has been increased to 60°. This creates an elliptical pattern that has twice the area as the one on the left, and the magnitude of illumination is decreased by half. The surface depicted on the right has an incident angle of 0°, but its distance from the light source is doubled relative to the one on the left. This causes the area of illumination to be increased by a factor of four, which reduces the magnitude of illumination by ¼ relative to the patch on the left.

**Figure 7.**
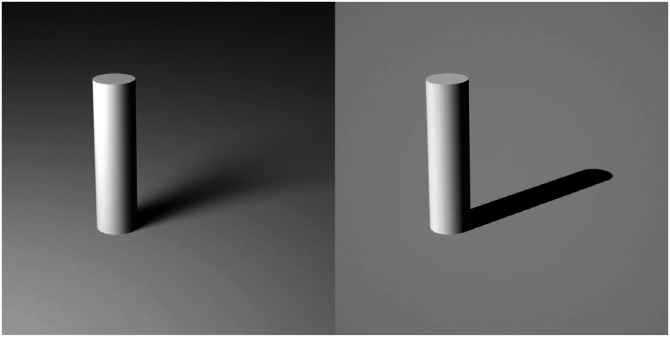
A cylinder illuminated by lights at different distances. The left panel shows a nearby source of illumination that produces an attenuation gradient along the ground plane. The right panel shows a distant source of illumination that produces uniform illumination along the ground plane. Note how this also affects the appearance of the cast shadows.

Let us now consider how these effects appear in a simple 3D scene. The left panel of Figure 7 shows an image of a ground surface with a single cylindrical column illuminated by an unseen light source to its left. Note that the pattern of shading along the ground has a clear attenuation gradient such that the shading is brightest in the lower left of the image and darkest in the upper right. This is the result of the inverse distance squared law of illumination. In addition, points on the cylinder that face toward the light source are brighter than points with a larger incident angle. This is the result of Lambert's cosine law of illumination.

One other thing to notice in this scene is that the pattern of illumination is inhomogeneous. The cylinder blocks the light from reaching some portions of the ground plane, which produces a cast shadow. The blurred part of the shadow is called a penumbra, and it occurs in regions where only part of the light source is occluded. The width of the penumbra is influenced by both the size of the light source and its distance from the illuminated object.

The right panel of Figure 7 shows exactly the same scene as the left panel, except that the light source has been moved much farther away and its power has been increased to simulate outdoor illumination from the sun. Since all points in the scene are a large distance from the light source, the variations in distance along the ground are quite small relative to the distance of the light source, and this effectively eliminates the attenuation gradient observed in the left panel. However, the gradient on the cylinder due to Lambert's cosine law remains largely unchanged. Note that the cast shadows in these scenes are quite different. The one produced by a distant light source has a very sharp edge, whereas the one produced by a nearby light source has a softer blurred boundary (i.e., a penumbra).

## Section 3: The Reflection of Light

**Figure 8.**
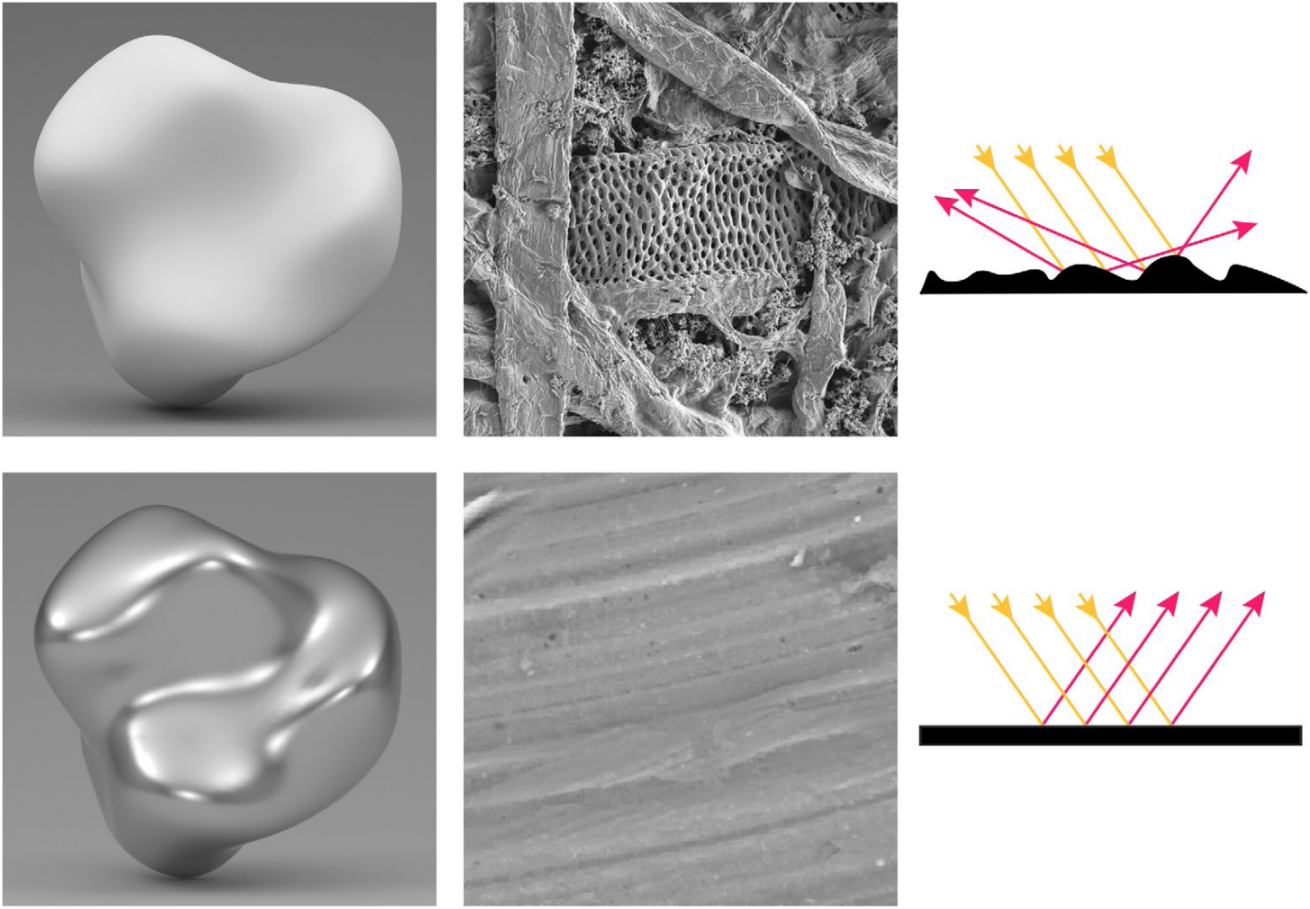
The left column shows computer rendered images of objects composed of office stationery (top) and polished metal (bottom). Both objects have the same pattern of illumination. The middle column shows these same materials as viewed through an electron microscope. These were photographed by Pavel Somov and Arlo James Barnes, respectively, and they are both licensed under CC-BY-4.0. The right column shows how light reflects from these surfaces. The incoming rays are colored orange, and the outgoing rays are colored red.

When light illuminates a surface, it can be reflected, transmitted, or absorbed in varying proportions depending upon the nature of the material. The pattern of reflection is influenced by the microscopic roughness of a surface. For example, the left column of Figure 8 shows computer rendered images of objects composed of office stationery (top) and polished metal (bottom) with same pattern of illumination. The middle column shows these same materials as viewed through an electron microscope. Although office stationery looks perfectly smooth to the naked eye at a macroscopic scale, its microscopic structure is composed of a complex 3D network of cellulous fibers. In contrast, the images of polished metal appear smooth at both scales. When a light ray reflects from a surface, the incident and exit angles are symmetrical about the surface normal in each local region. If the microscopic surface structure is smooth and flat, then incident rays that are parallel to one another will also be reflected in parallel (see the lower right panel). However, if the microscopic structure is sufficiently rough, then incident rays that are parallel to one another will be scattered in different directions (see the upper right panel).

Lambert's research was primarily focused on microscopically rough surfaces that scatter light uniformly in all directions, and this type of uniform scattering is now referred to as Lambertian reflectance. The upper left panel of Figure 8 is an example of this type of material. Note that it appears matte (as opposed to shiny). The pattern of shading on a Lambertian surface varies systematically with the direction of illumination and is completely independent of the direction of view.

**Figure 9.**
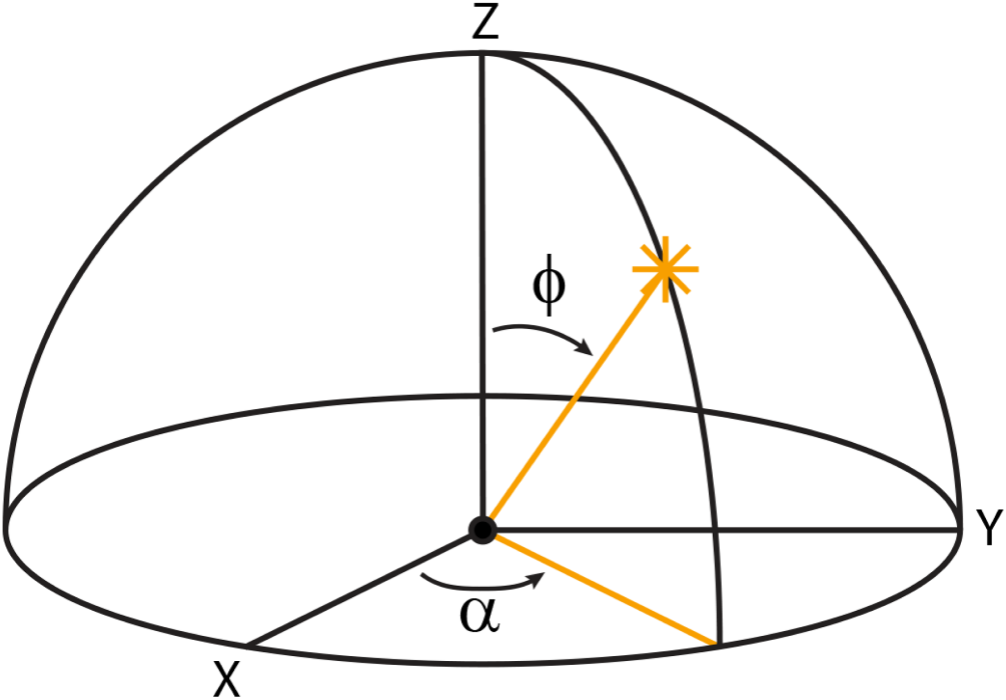
Azimuth and polar angles of the direction of illumination. The azimuth (*a*) can be thought of as the orientation of a sun dial relative to some reference axis on a surface (e.g., north). The polar angle (*f*) is the angle of the light ray relative to the surface normal.

The 3D orientation of a light ray has two degrees of freedom, which can be parameterized in a variety of ways. One common approach is to represent the orientation in terms of its azimuth and polar angles (see Figure 9). The azimuth (*α*) can be thought of as the orientation of a sun dial relative to some reference axis on a surface (e.g., north). The polar angle (*ϕ*) is the angle of the light ray relative to the surface normal. The intensity of a reflected ray on a Lambertian surface is invariant over changes in the azimuth of illumination, and it varies as a cosine function of the polar angle.

### Bidirectional Reflectance Distribution Functions

**Figure 10.**
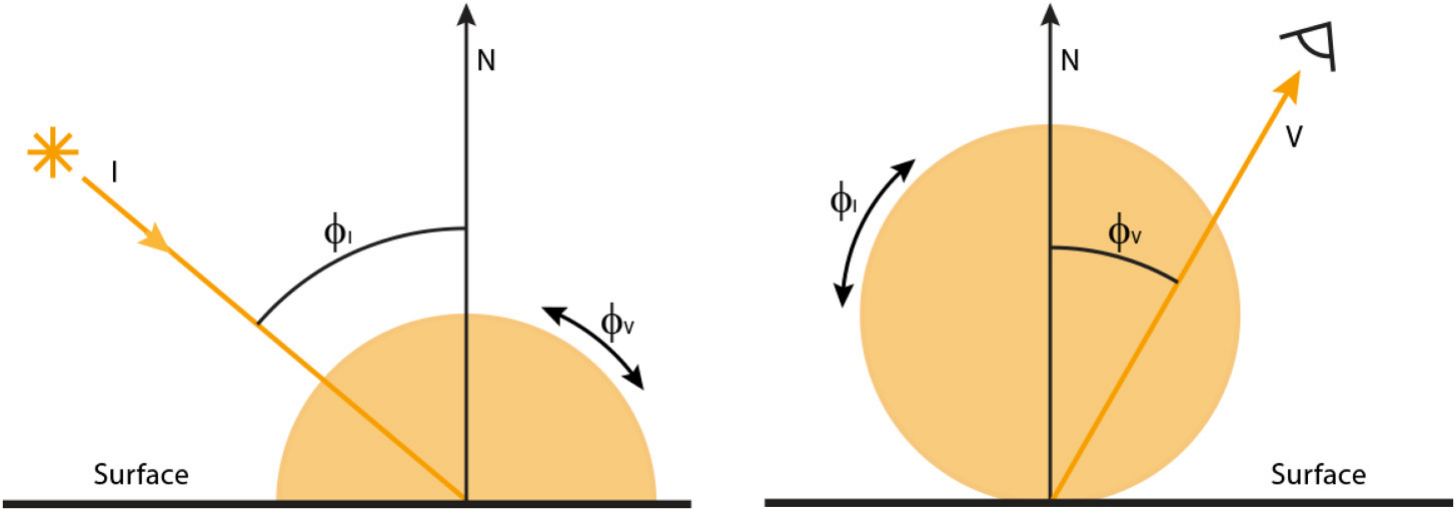
A 2D graph of a bidirectional reflectance distribution function for a surface material that scatters light uniformly in all directions. In the left panel, the polar angle of illumination (*f*_I_) is fixed, and the polar angle of the viewing direction (*f*_V_) varies over a 180° range. In the right panel, the viewing direction is fixed, and the polar angle of illumination varies. See text for details.

It is important to keep in mind, however, that many surfaces in the natural environment do not scatter light uniformly in all directions. In general, the light that reflects toward the point of observation can be influenced by both the azimuth and polar angles of the light source, as well as the azimuth and polar angles of the viewing direction. Thus, the pattern of reflectance off any given surface region is a function of four variables called the bidirectional reflectance distribution function (BRDF). Since the structure of that pattern is difficult to visualize, it is often useful to simplify it by holding three of the variables constant and plotting the remaining one in a 2D graph. This is typically achieved in two different ways. One is to plot the luminance for each possible polar angle of the viewing direction (*ϕ*_V_) for a fixed polar angle of illumination (*ϕ*_I_), as shown in the left panel of Figure 10. The luminance for any given viewing direction is represented by the distance in that direction from the surface point to the boundary of the orange region. For surface materials that scatter light uniformly in all directions, the luminance remains constant over all possible viewing directions and the BRDF is represented as a semicircle.

An alternative approach shown in the right panel is to plot the luminance for each possible polar angle of illumination (*ϕ*_I_) for a fixed polar angle of the viewing direction (*ϕ*_V_), as shown in the right panel of Figure 10. This representation is a bit less intuitive than the one on the left because all points along the orange boundary represent luminance in the same viewing direction. It is the illumination angle that is being varied. For any given direction of illumination, the luminance is represented by the distance in that direction from the surface point to the boundary of the orange region. For a Lambertian BRDF, the luminance changes as a cosine function of the polar angle of illumination. Luminance is at a maximum when the illumination is perpendicular to the surface, and it approaches zero as the illumination becomes parallel to the surface. Thus, the BRDF is represented as a circle.

**Figure 11.**
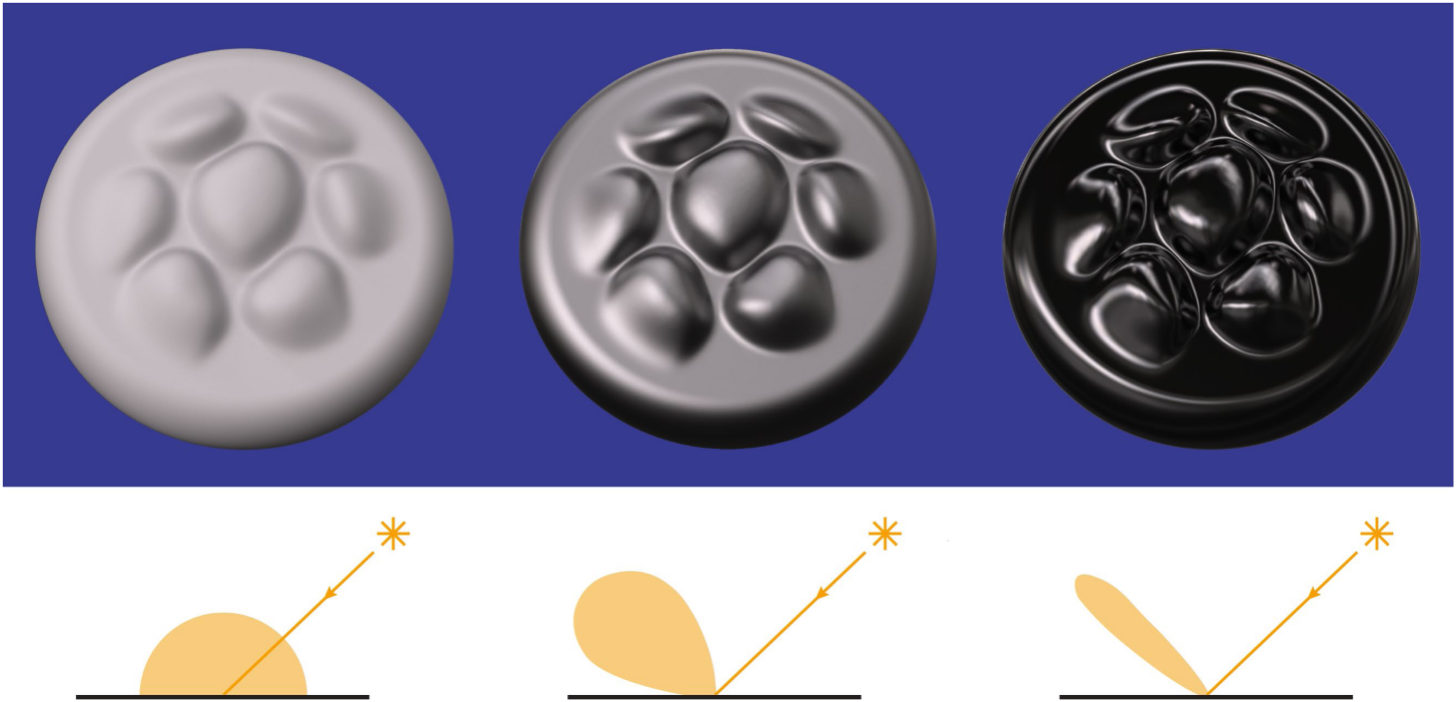
The effects of microscopic roughness on surface reflections and how that affects the BRDF. Left: A Lambertian surface that scatters light uniformly in all directions and is perceived as matte white. Right: A surface that produces specular reflections with negligible scattering and is perceived as shiny black. Middle: A surface whose roughness is in between purely specular and Lambertian that is perceived as slightly glossy. The pattern of shading for Lambertian materials is only influenced by the directions of illumination, whereas the shading for non-Lambertian materials is also influenced by the direction of view.

Figure 11 shows the images of three surfaces that vary in terms of their microscopic roughness and the diagram below each image shows the BRDF of the depicted material. The surface on the left reflects light in a manner that is similar to office stationery such that illumination is scattered diffusely in all possible directions as shown in the top row of Figure 8. What we perceive as the lightness of an object is most often associated with its diffuse reflections. Reflectance (or albedo) is defined as the proportion of incident light that is reflected. For example, surfaces perceived as white typically reflect a large proportion of the incident light and absorb the rest. Surfaces perceived as black reflect a small proportion of the incident light.

The right panel of Figure 11 shows the same object as in the left panel with the same pattern of illumination, but the microscopic structure of the surface is much smoother. Microscopically smooth surfaces produce mirror (or specular) reflections of the surrounding environment, and they are responsible for the perception of gloss. When combined with the absence of diffuse reflections, the image appears as a shiny black surface. The bright spots on shiny surfaces are referred to as specular highlights and they are primarily caused by reflections of light sources. Another important aspect of shiny materials is that the locations of highlights on a surface change with the direction of view, unlike Lambertian shading. Specular reflections are the greatest when the illumination and viewing angle are symmetrical about the surface normal, and it drops off rapidly as they deviate from that.

Most of the materials we encounter in the natural environment are somewhere in between perfect mirrors and perfect scatterers as shown in the middle panel of Figure 11. As the amount of scattering increases relative to a perfect mirror, the specular highlights become more and more blurred. These surfaces may still appear glossy, but the appearance of gloss will eventually disappear as the scattering gets closer to Lambertian reflectance.

**Figure 12.**
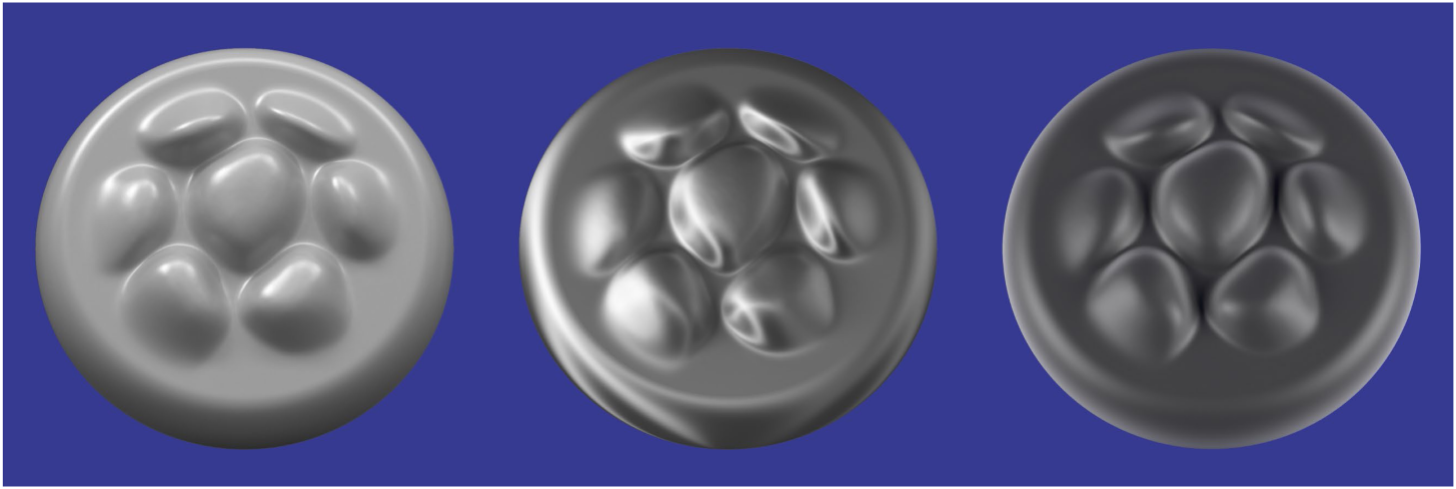
The patterns of shading for three materials that produce a linear combination of diffuse and specular reflections. Left: This surface combines a matte material with a shiny transparent coating such as shellac. Middle: A surface composed of microgrooves in a single dominant direction, such as satin, whose reflections are influenced by the orientation of the incident light relative to the microgrooves. Right: A surface that contains short shiny fibers like velvet or peach that produce grazing angle highlights.

It is also possible for surface materials to produce a linear combination of diffuse and specular reflections. This can be achieved by covering a matte material with a smooth transparent coating such as shellac as shown in the left panel of Figure 12. Some light is reflected off the transparent coating in a specular manner, while other light is transmitted through the coating and reflected diffusely by the underlying opaque material. The BRDF for a coated surface is a linear combination of the ones shown in the left and right panels of Figure 11.

The middle panel of Figure 12 shows another way of combing diffuse and specular reflections that occurs on surfaces such as woven silk or satin with microgrooves in one or more dominant directions. These surfaces produce anisotropic reflections in which the scattering of light varies as a function of the azimuth angle of illumination. Incident light that is parallel to the grooves will produce specular reflections, but light that is perpendicular will be scattered. This cannot be represented with a 2D plot of the BRDF. If the azimuth of illumination is held constant in one direction, then the BRDF will resemble the one for Lambertian reflection in the left panel of Figure 11, but if the azimuth is in the orthogonal direction, then the BRDF will resemble the one for specular reflection in the right panel of Figure 11.

The right panel of Figure 12 shows a material like black velvet cloth that is composed of a dark matte base in which short fibers are embedded (Koenderink & Pont, 2003). Illumination that is perpendicular to the surface will be primarily influenced by the base material, but illumination at high incident angles will be reflected off the embedded fibers to produce grazing angle highlights. The BRDF in that case is the opposite of Lambertian reflectance (Koenderink & Pont, 2003). The luminance is the darkest when the direction of illumination is perpendicular to the surface and the brightest when it is close to parallel.

### Constraints on Image Shading

**Figure 13.**
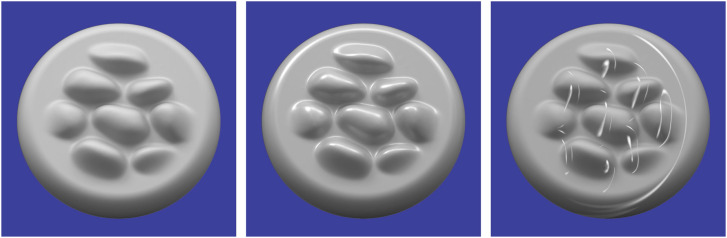
Left: A bumpy surface depicted with diffuse shading that appears matte. Middle: The same surface depicted with a linear combination of diffuse and specular shading that appears glossy. Right: An image that is identical to the one in the middle panel, except that the specular highlights have been rearranged on the surface so that they are no longer aligned with the directions of minimum curvature. Note how that manipulation eliminates the perception of glossiness.

Let us now consider how the reflection of light constrains the patterns of shading within a visual image. For diffuse reflections, the brightest points in an image are those that project to points on a surface where the local surface orientation is facing the light source. Surfaces that are uniformly convex will contain a single point that satisfies that criterion, and images of those surfaces will have a single local maximum of intensity. Surfaces with varying signs of curvature will contain multiple points that face toward the direction of illumination, and the shaded images of those surfaces will have multiple local maxima of intensity. Due to this relationship between the number of surface points that face toward the light source and the number of local maxima in a shaded image of that surface, there is a surprisingly tight correspondence between the qualitative 3D structure of a surface and the structure of its shaded image. Note that this is clearly evident in the left panel of Figure 13. Each of the bumps depicted in that image has the same basic pattern of shading that is brighter on top than on the bottom, thus indicating that the illumination is coming from above the line of sight.

For specular reflections, the brightest points in an image correspond to surface points where the normals bisect the angle between the viewing and illumination directions. As the surface normals deviate from those specific orientations, the intensities of the specular reflections drop off rapidly. This also produces a tight correspondence between the qualitative 3D structure of a surface and the structure of its shaded image, which can be observed in the middle panel of Figure 13. Note that each bump contains a single specular highlight, though there are also additional highlights along the concave regions at the base of each bump.

Since the intensity of specular reflections change rapidly as a function of surface orientation, the specular highlights on an object tend to align along directions of minimal curvature. The object depicted in the middle panel of Figure 13 contains bumps whose directions of minimal curvature are all oriented in a horizontal direction, and the specular highlights are all aligned horizontally as well. This makes the surface appear to have a high level of gloss. However, if the specular highlights are rearranged on the surface so that they are no longer aligned with the directions of minimum curvature as shown in the right panel of Figure 13, then the appearance of gloss is almost entirely eliminated. This phenomenon was first discovered by Beck and Prazdny (1981), and a more systematic investigation of the effect was later performed by Anderson and Kim (2009).

### The Effects of Color

All of the effects described thus far can be modeled using geometrical optics, but the appearance of color requires a consideration of the wavelengths of light. The color of light that stimulates receptors on the retina is influenced by two factors: One is the distribution of wavelengths for the sources of illumination within a scene, and the second is the reflection and absorption characteristics of visible surfaces. The proportion of light reflected from a surface often varies as a function of wavelength. For example, surfaces perceived as red reflect long wavelengths of light within the visual spectrum and absorb shorter wavelengths. Surfaces perceived as blue reflect short wavelengths and absorb longer ones. However, these surfaces can only reflect wavelengths that are present within the pattern of illumination. Thus, a red surface illuminated by blue light may not reflect any light at all.

**Figure 14.**
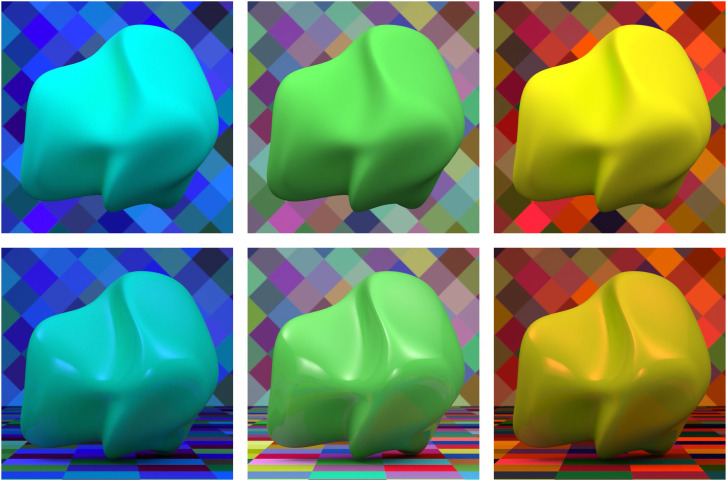
The top row shows three images of a green object against a background with randomly colored diamonds. The reflectance properties are identical in all three scenes, but the illumination is different. Moving from left to right, the illumination is primarily blue, white, and primarily red. The bottom row shows the same three scenes as in the top row with the same patterns of illumination. However, the depicted green objects have a clear transparent coating that produces specular highlights.

The top row of Figure 14 shows three computer rendered images of a green Lambertian object against a background with randomly colored diamonds. The surface reflectance properties are identical in all three scenes, but the illumination is different. Moving from left to right, the illumination is primarily blue, white, and primarily red. Note that the apparent color of the green surface changes with the pattern of illumination. That is to say, the green object with blue light is perceived as cyan, the one with white light is perceived as green, and the one with red light is perceived as yellow.

The bottom row of Figure 14 shows the same three scenes as in the top row with the same patterns of illumination. However, the depicted green objects have a clear transparent coating that produces specular highlights. Since the light that reflects off the coating does not interact with the underlying surface material, the color of the specular highlights is only affected by the pattern of illumination. In principle, this could provide information to determine the true color of the underlying surface (Xiao et al., 2012; Yang & Maloney, 2001; Yang & Shevell, 2003). Note that the shiny green objects under red or blue illumination appear slightly more green than the comparable matte objects in the top row, but this effect is relatively weak.

### Psychological Research on the Effects of Reflection

There is a large body of research that has examined how patterns of reflection influence human perception. It is important to keep in mind that surface reflectance is a physical property that describes the proportion of incident light that is reflected. Lightness is a psychological property that describes the perception of reflectance. A comprehensive review of the literature on lightness constancy can be found in Gilchrist (2006) and a review of the literature on color constancy is provided by Foster (2011). In addition to the research on lightness and color constancy, there is another substantial literature on the perception of gloss (e.g., see Anderson & Kim, 2009; Marlow & Anderson, 2013). Reflectance distribution functions also play an important role in the perception of 3D shape from shading. Much of the research in that area has been limited to surfaces with Lambertian reflectance, but more recent studies have shown that judgments of 3D shape remain remarkably stable over a wide range of different reflectance functions (see Todd et al., 2014, 2023).

## Section 4: The Transmission of Light

Another thing that can happen when light illuminates a surface is that it can be transmitted inside the material. When light crosses a boundary between two materials at an oblique angle, its direction changes, which is referred to as refraction (see Figure 1). This is characterized by a dimensionless number called the index of refraction (IOR) that defines the magnitude of direction changes for any given material relative to a vacuum. The IOR is a complex number. The real part (*n*) is based on the speed of light in a material, whereas the imaginary part (*k*) is based on the propensity of a material to absorb light. This imaginary component is also referred to as the extinction index or the coefficient of extinction. The website https://refractiveindex.info provides a convenient resource to obtain the measured values of *n* and *k* for a wide variety of natural and man-made materials. For nonmetallic materials, the value of *k* is almost always close to zero, so that the refraction of light in those materials is completely determined by *n*. For example, the values of *n* for water, glass, and diamond are 1.33, 1.50, and 2.40, respectively. These values indicate that the bending of light is much larger for diamond than for water or glass.

**Figure 15.**
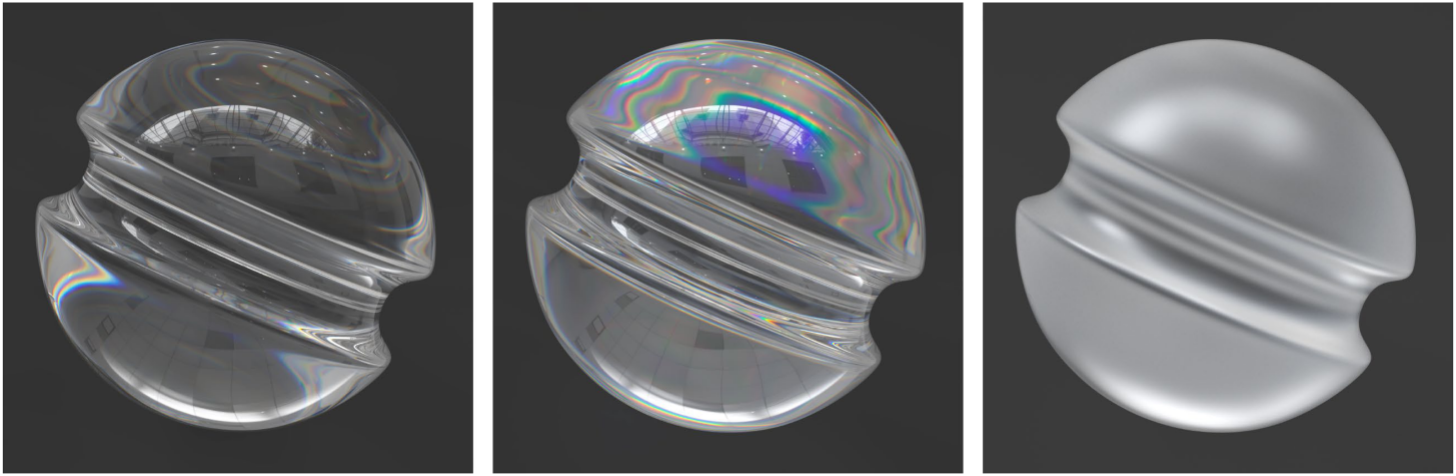
(Left) An object composed of a K7 crown glass material with a high Abbe number. (Middle) An object composed of a Swarovski (sf66) lead crystal material with a low Abbe number (Right). The same material as in the middle panel with a roughened surface.

Since the refraction of light can vary as a function of wavelength, the values of *n* are typically measured at a standard wavelength of 589 nm. When light is transmitted through particles of water in the air, it is the variation of *n* with wavelength that produces the appearance of rainbows. This effect is referred to as chromatic dispersion, and it is typically measured for any given material as a ratio of the refractive indices at different wave lengths, called the Abbe number. Materials with high Abbe numbers produce small amounts of chromatic dispersion relative to materials with low Abbe numbers. The Maxwell renderer comes with a library of materials with measured values of the complex IOR at multiple wave lengths. For example, the image in the left panel of Figure 15 was created using a K7 crown glass material with a high Abbe number. Note that the glass is mostly clear, except for a small amount of dispersion just below the valley on each side. The image in the middle panel depicts a Swarovski (sf66) lead crystal material with a low Abbe number. It produces a much larger amount of dispersion that covers the entire upper half of the object.

The right panel of Figure 15 shows the same lead crystal material with a roughened surface, which is often referred to as frosted glass. When light hits the boundary of that surface, it is transmitted inside the glass, but the directions of the transmitted rays are scattered randomly in different directions. This gives the material a translucent appearance that is much different from the perception of smooth glass. It also eliminates the effect of chromatic dispersion.

### Thin Film Interference

There is another aspect of physical optics in addition to chromatic dispersion that can also produce color in clear transparent materials. This occurs when light is transmitted through very thin layers. Some light reflects off the front surface of the layer, and other light is reflected off the back surface. If the thickness of the layer is just right, the two patterns of reflection will interfere with one another, producing positive interference at some wavelengths and negative interference at others. This is sometimes referred to as iridescence and it is what causes the appearance of color on soap bubbles.

**Figure 16.**
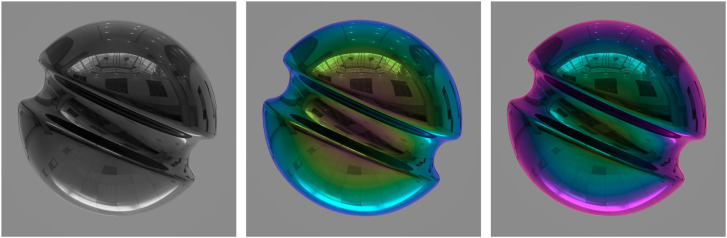
Three objects with identical shapes and patterns of illumination. They are all composed of a black Lambertian material that is covered with a smooth transparent coating. The only difference among the three panels is the thickness of the smooth coating. From left to right, the simulated thicknesses are 50,000 nm, 500 nm and 450 nm, respectively. The differences in color are caused by thin film interference.

For example, the left panel of Figure 16 shows a matte black surface with a transparent coating that has a thickness of 50,000 nm (.05 mm). Note that the specular reflections are all white. The middle panel of Figure 16 shows what happens if the thickness of the coating is reduced to 500 nm. The light waves reflecting off the top and bottom surfaces of a thin film interfere with one another, which creates a spectrum of different colors. The surface depicted in the right panel of Figure 16 has a coating with a thickness of just 450 nm, and it produces a different spectrum of colors. It is important to keep in mind that all of these images were generated with exactly the same surface materials and lighting. All that differs between them is the thickness of the transparent coating.

### Refractive Distortions of Background Surfaces

**Figure 17.**
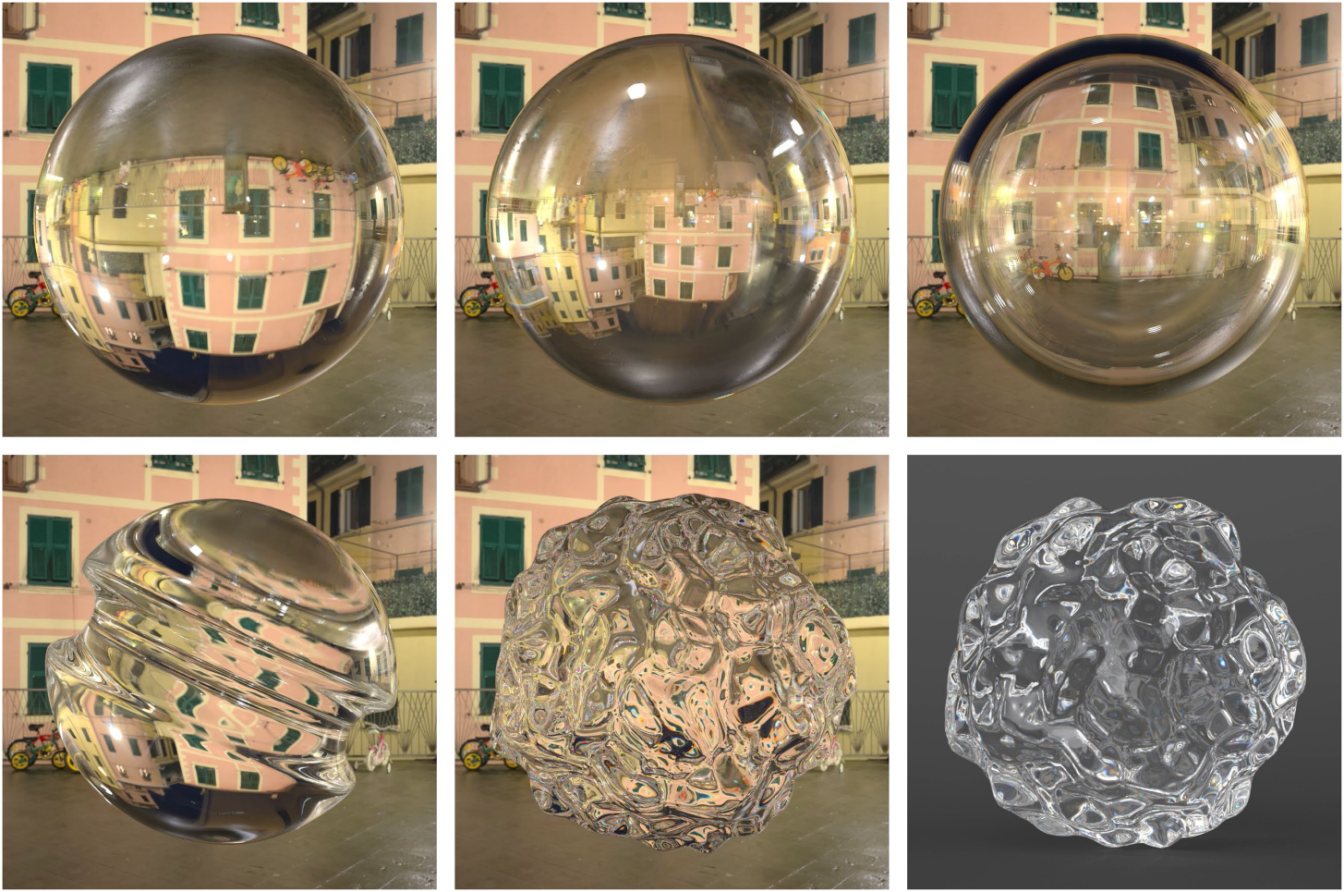
The top row shows three images of a transparent sphere presented in front of an Italian piazza. From left to right they depict solid glass, solid diamond, and hollow diamond materials. The bottom row shows three glass objects with more complex shapes. The one on the right is identical to the one in the middle, except it is presented against a homogeneous gray background.

One of the most salient aspects of refraction is that it distorts the appearance of background surfaces that are seen behind a transparent material. One factor that influences the amount of distortion is the refractive index of the transparent material (see Fleming et al., 2011). The top row of Figure 17 shows images of transparent spheres presented in front of an Italian piazza. The one on the left depicts a solid glass sphere, and the one in the middle depicts a solid diamond sphere. Note that the diamond material produces much larger refractive distortions than glass, and that the background appears upside down in both images. The top right panel of Figure 17 shows a hollow diamond sphere. The refractive distortions are much smaller in that case and the background appears right side up. This also provides a more compelling impression of a transparent material.

Another important factor that influences the amount of refractive distortion is the pattern of curvature on the transparent surface. For example, when looking through a glass window, the scenes behind the glass appear completely undistorted, but that is not the case for more complex 3D objects. The bottom left panel of Figure 17 shows a yoyo shaped object presented in front of the same piazza as in the top row. The object is mostly spherical, except that it has a concave valley that circles around its center. This added concavity causes the refractive distortions to be much more severe than those that occur for uniformly spherical shapes. The middle panel of the bottom row shows a bumpy sphere in the same Italian piazza. The refractive distortions are so severe in that case that they have almost no resemblance to the background scene, and it detracts from the appearance of transparency. However, when this same object is presented against a homogeneous dark background as shown in the right panel, the appearance of transparency is much more compelling. This is probably why professional photographers almost never photograph glass objects against cluttered backgrounds.

### Subsurface Scattering

**Figure 18.**
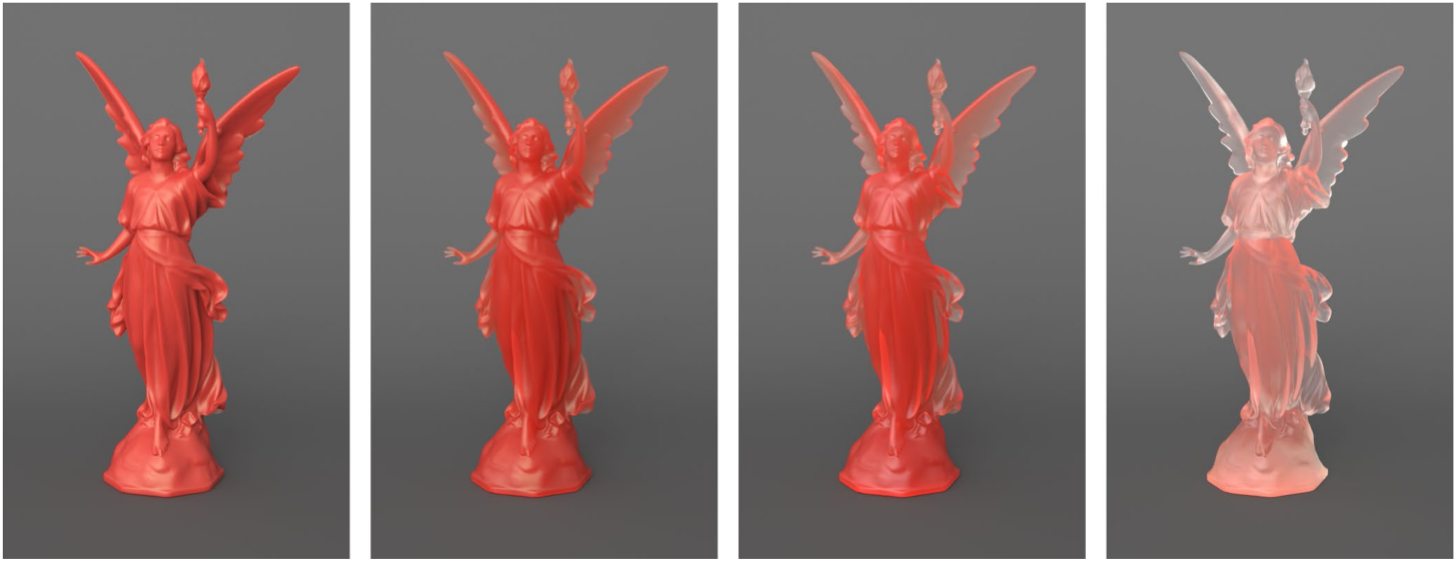
Four objects with identical shapes and patterns of illumination. The one the left depicts a material like porcelain that has a high degree of scattering, but light can only penetrate a short distance inside the material. The one the right depicts a material like honey that produces relatively little scattering and light can penetrate a large distance inside the material. The two images in the middle show intermediate combinations of these parameters.

Glass is a special material because it allows the transmission of light without any of it being scattered. For other materials that transmit light like milk or wax, the light becomes scattered by molecules inside the material. There are two key parameters that influence the behavior of light within the volume of an object. One is the distance that light can penetrate before it is absorbed, and the other is how much the light is scattered. For example, the image in the left panel of Figure 18 depicts a material like porcelain that has a high degree of scattering, but light can only penetrate a short distance inside the material. The one in the right panel, in contrast, depicts a material like honey that produces relatively little scattering and light can penetrate a large distance inside the material. The two images in the middle show intermediate combinations of these parameters. Lambertian surfaces and clear glass can be thought of as the endpoints of this continuum. Lambertian materials allow negligible penetration and produce uniform scattering. Glass, on the other hand, allows large amounts of penetration with negligible scattering.

**Figure 19.**
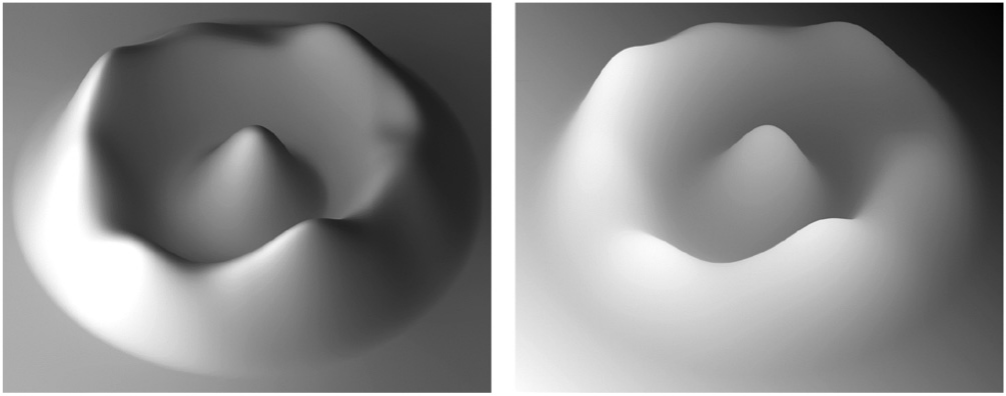
A surface depicted with Lambertian shading that is perceived as opaque (left), and one that was textured with a linear intensity ramp that is perceived as translucent (right).

Marlow et al. (2017) have pointed out that luminance from opaque surfaces covaries with surface orientation, but that the luminance from translucent surfaces is largely independent of surface orientation. To investigate this as a useful source of information, they showed that an opaque surface can be made to appear translucent, and a translucent surface can be made to appear opaque by manipulating the apparent 3D shape of an object or its pattern of illumination. A similar demonstration by Todd et al. (2014) is shown in Figure 19. The surface depicted on the left has a Lambertian reflectance function, in which luminance varies as a cosine function of the angle between the surface normal and the direction of illumination. The surface depicted on the right, in contrast, was textured with a planar projection of a linear intensity gradient, so that the local image intensity in each region is based on its position irrespective of its orientation. Note that the surface on the left appears opaque, whereas the one on the right appears translucent.

### Psychological Research on the Effects of Light Transmission

Most classical research on the perception of transparency has focused on overlapping planar surfaces, and an excellent review of that work is provided by Singh and Anderson (2002). More recent research involving colored 3D objects has been reported by Ennis and Doerschner (2021). Visual information for the perception of glass has been investigated by Todd and Norman (2019, 2020). There have also been numerous papers on the perception of translucency from subsurface scattering including Fleming and Bülthoff (2005), Xiao et al. (2014), and Marlow et al. (2017).

## Section 5: Fresnel Effects

As we discussed earlier, illumination is the magnitude of incident light on a surface per unit area, and reflectance is the proportion of illumination that is reflected. Illumination varies as a cosine function of the incident angle because that is the function that determines the area over which the light is distributed. For the diffuse components of the BRDF, the angle of illumination has no effect on reflectance. However, that is not generally the case for the specular reflections of shiny surfaces.

**Figure 20.**
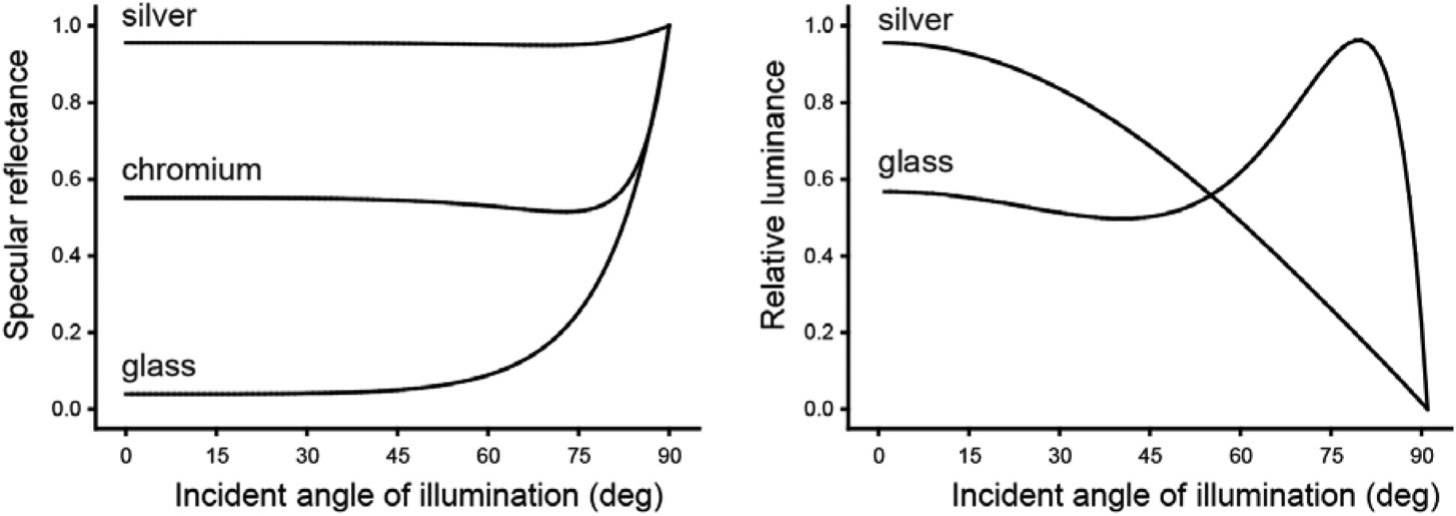
Specular reflectance and relative luminance as a function of the incident angle of illumination for metals and dielectric materials. Note that silver (a metal) has the highest luminance at small incident angles, whereas glass (a dielectric) has the highest luminance at large incident angles.

The materials encountered in the natural environment can be grouped into two optical categories: those that conduct electricity, called metals (e.g., silver, gold, or copper); and those that do not conduct electricity, called dielectrics (e.g., glass, plastic, or wood). The left panel of Figure 20 shows the specular reflectance of three natural materials as a function of the polar angle of illumination. These include two metals (silver and chromium) and one dielectric (glass). Note in particular how the effect of incident angle varies dramatically among different materials. For silver, almost all illumination is reflected at all incidence angles. For glass, on the other hand, very little light is reflected except at high incidence angles. The increase in specular reflectance at high incident angles, especially for dielectric materials, is often referred to as the Fresnel effect in honor of the French physicist Augustin-Jean Fresnel who first discovered it.

A key parameter that determines the shapes of the curves in the left panel of Figure 20 is the index of refraction, which is a complex number (*n*, k). Although the imaginary component (k) is negligible for dielectric materials like glass, it has a significant impact on the reflection of light for metals. The IORs for silver, chromium, and glass are (.05, 3.92), (3.21, 3.30), and (1.5, 0), respectively, at a wavelength of 589 nm. These values can change significantly for different wavelengths, which can alter the shapes of the curves shown in Figure 20. The surface reflectance can also be affected by the direction of polarization of the incident light, although most of the light encountered in natural vision is unpolarized.

It is important to keep in mind that the light reflected toward the point of observation (i.e., luminance) is a product of reflectance and illumination, and that illumination varies as a cosine function of the incidence angle, which is the geometric basis for Lambert's law. Thus, it is possible to compute the luminance of specular reflections by multiplying the reflectance values derived from the Fresnel equations times the cosine of the incident angle. The right panel of Figure 20 shows the relative luminance as a function of the incident angle for both glass and silver. These curves have been normalized to compensate for the large difference in the magnitude of specular reflections for these materials. Note that the luminance varies as a cosine function of the incident angle for silver, but that the effect of incident angle is more complex for glass. The maximum luminance in that case occurs at an incident angle of 79°.

**Figure 21.**
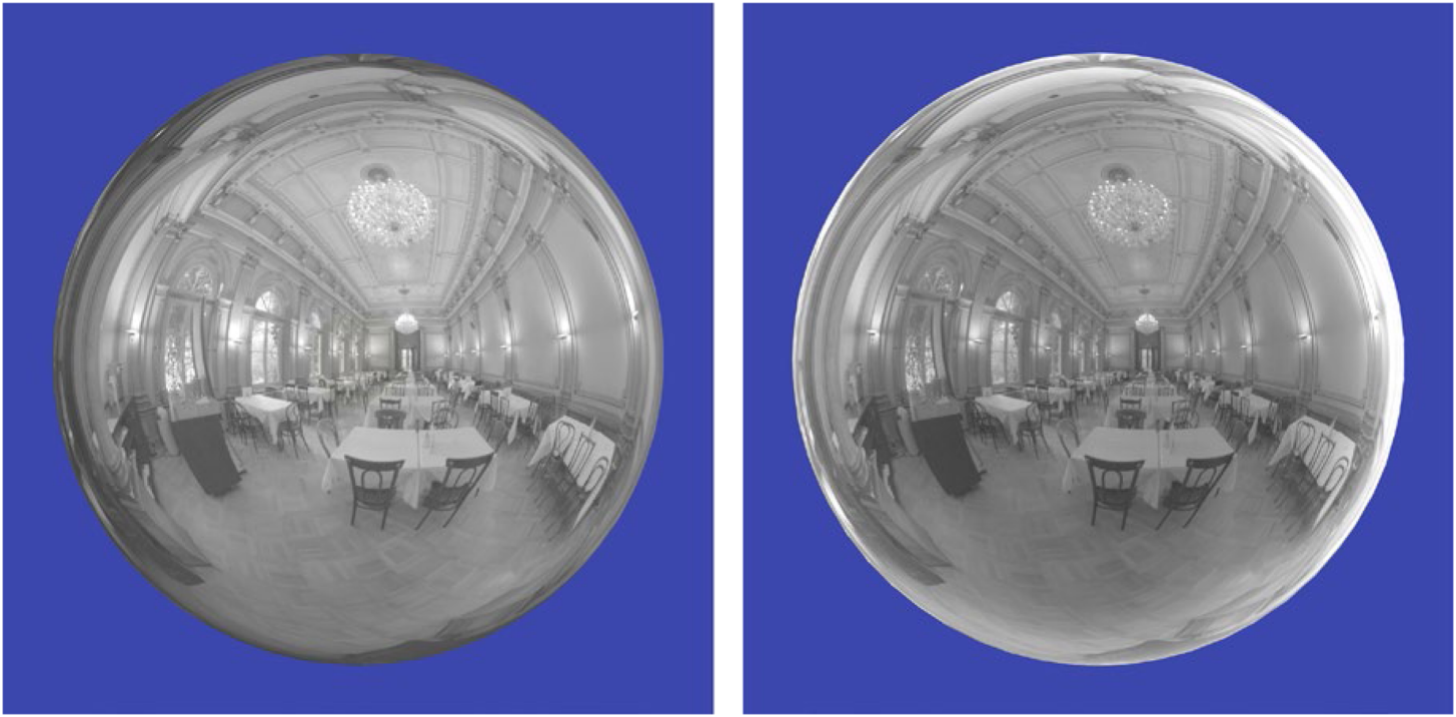
Specular reflections of a silver sphere (left) and an obsidian glass sphere (right). Note how the reflections are the brightest in the center for silver, and the brightest in the periphery for obsidian.

Figure 21 shows two spherical objects illuminated by a light map of a banquet hall. The image on the left depicts a silver material with zero roughness. The one on the right depicts an obsidian surface (i.e., volcanic glass) with zero roughness. Both of these were created using the Maxwell material library that uses measured values of the complex IOR at different wavelengths. Since silver reflects a much higher proportion of the incident light than glass, the exposure of the glass image has been increased appropriately so that both images have the same mean luminance. Despite that normalization, however, the two images appear noticeably different. Note that the silver reflections are the brightest in the center of the sphere and become darker in the periphery. The obsidian reflections, in contrast, are the darkest in the central regions and brighter in the periphery due to the Fresnel effect. This is the pattern of specular reflection that is typically observed on nonmetallic shiny objects in the natural environment.

**Figure 22.**
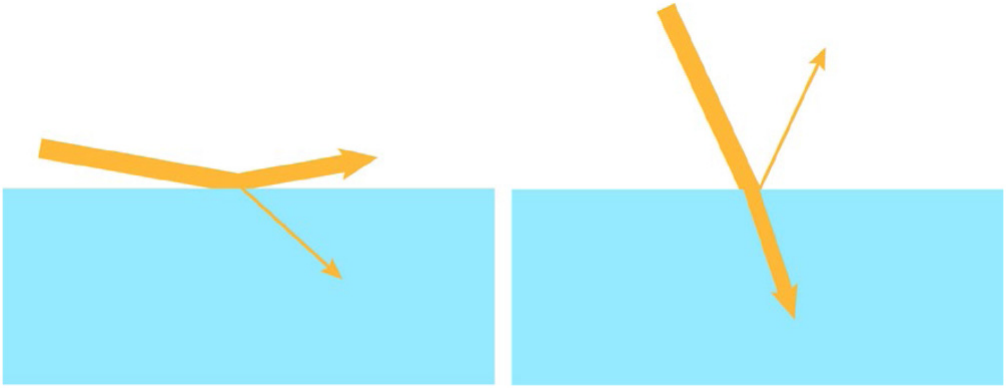
When light illuminates a surface at a high incident angle as shown in the left panel, most of it is reflected and relatively little is transmitted. Conversely, when light illuminates a surface at a high incident angle as shown in the right panel, most of it is transmitted and relatively little is reflected.

The light that is not reflected from the surface of an object is transmitted inside the material. For some opaque materials like high gloss black paint, the transmitted light is absorbed and transformed into thermal energy. For other transparent materials like glass or water, the light propagates through a new medium after undergoing refraction. Due to the Fresnel effect, the incident light is mostly reflected at high illumination angles, and mostly transmitted at small illumination angles (see Figure 22).

**Figure 23.**
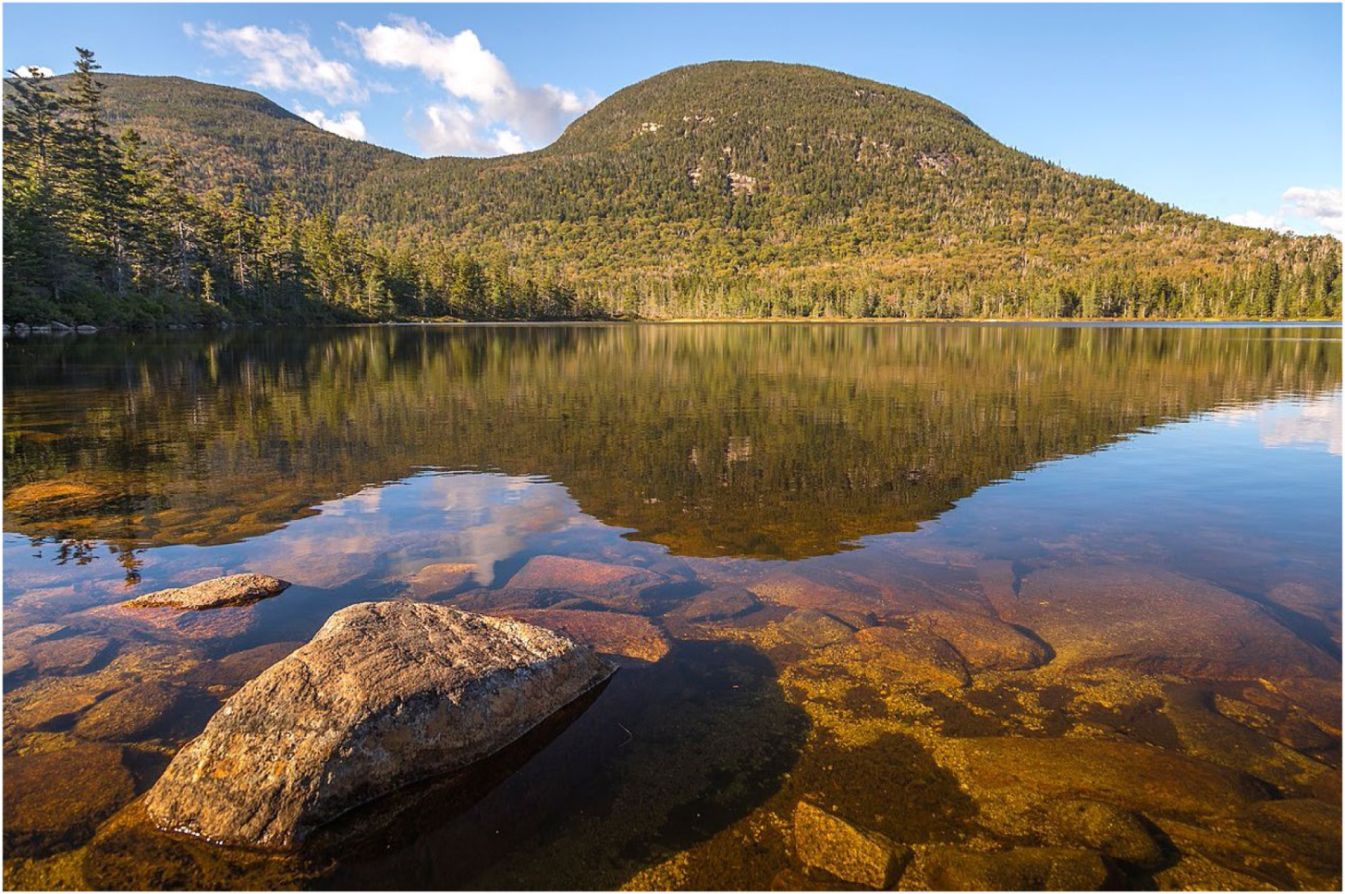
A photograph by Christian Collins, licensed under CC-SA-2.0. In the nearest parts of the lake, rocks are visible underneath the surface of the water, but all that is visible in the farthest parts are the reflections of the green mountains and the blue sky.

These effects are frequently observed when viewing a shallow pool of water as shown in Figure 23. The light that reaches the eye from the nearest part of the lake strikes the surface of the water at a small incident angle. Thus, most of it is transmitted, making it possible to see the rocks below the surface. The light that reaches the eye from the farthest part of the lake strikes the surface of the water at a large incident angle. Most of that light is reflected, so the reflections of the green mountains and the blue sky are perceptually prominent. Note that there is a region of transition about one-third up from the bottom where the reflected and transmitted light can be observed simultaneously.

**Figure 24.**
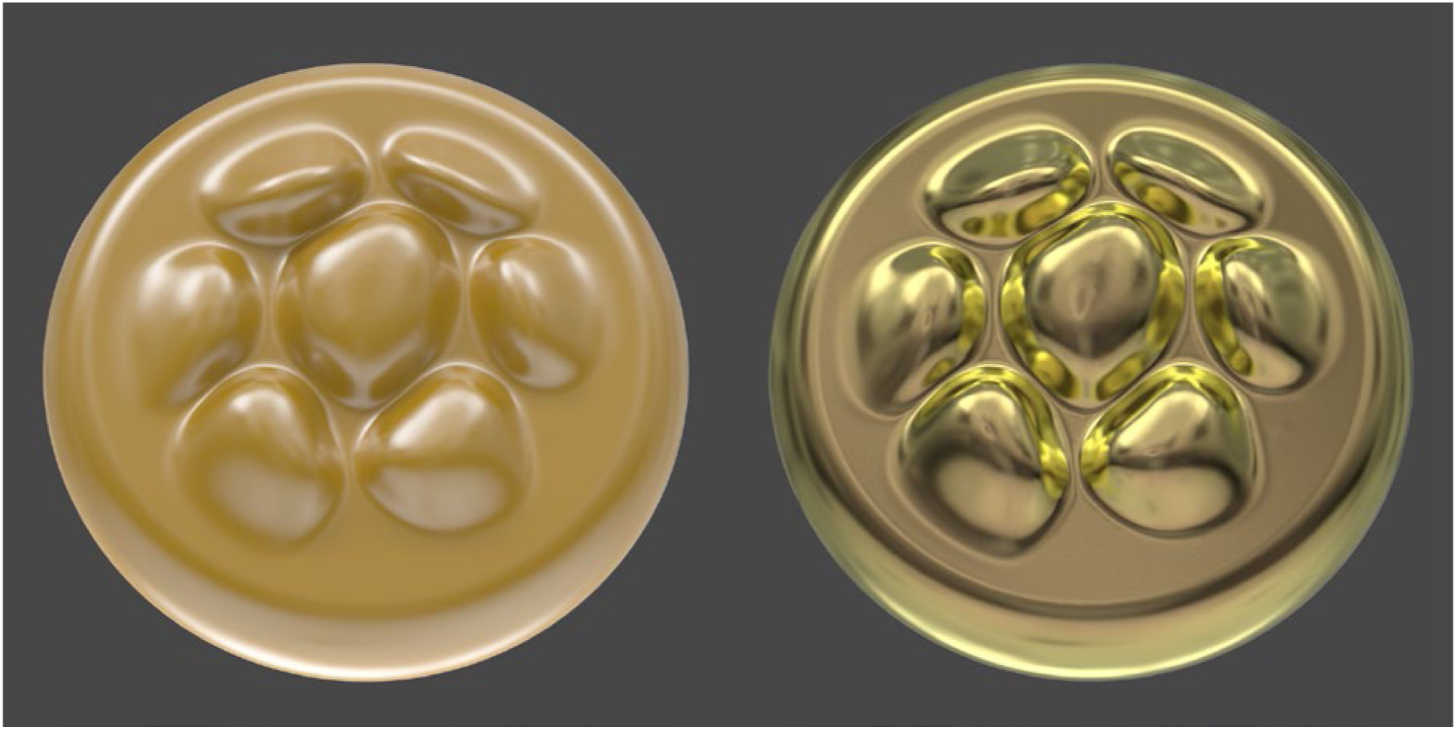
Two materials composed of gold colored plastic (left) and gold metal (right). Note that the specular reflections represent the color of the light for plastic and the color of the material for metal.

The complex IOR also determines how the wavelengths of reflections change as a function of the incident angle, and that is an important factor for simulating the colors of metal materials. For example, consider the object depicted in the left panel of Figure 24. It is composed of a gold colored matte material covered with a smooth transparent coating. Note that the diffuse reflections are colored gold from the underlying matte material, but the specular reflections from the transparent coating represent the color of the white light source. The right panel of Figure 24 shows the same geometric shape as in the left panel with the same pattern of illumination, but the depicted material is gold metal as opposed to gold colored plastic. For metals, 100% of the reflected light is specular, but the color of those reflections can be influenced by the surface material. This is why the specular reflections of the object in the right panel appear yellow or gold, even though the illumination is white.

### Psychological Research on the Fresnel Effects

Recent experiments by Todd and Norman (2018, 2019, 2020) and Norman et al. (2020) have investigated the perceptual categorization of metal and dielectric materials, but those studies were limited to monochromatic stimuli. It remains to be determined how these judgments are influenced by chromatic stimuli like the ones shown in Figure 24.

## Section 6: Indirect Illumination

When light is reflected off one surface, it can provide a source of illumination for another, and the reflected light from that surface can also illuminate other surfaces. These surface-to-surface reverberations of light energy are referred to as interreflections, and the illumination of one surface by reflections from another is called indirect (or global) illumination. The only thing that prevents this process from continuing indefinitely is that some energy is absorbed on each bounce. Thus, surfaces with low reflectance produce a smaller number of indirect bounces than surfaces with high reflectance (see Gilchrist & Jacobsen, 1984).

**Figure 25.**
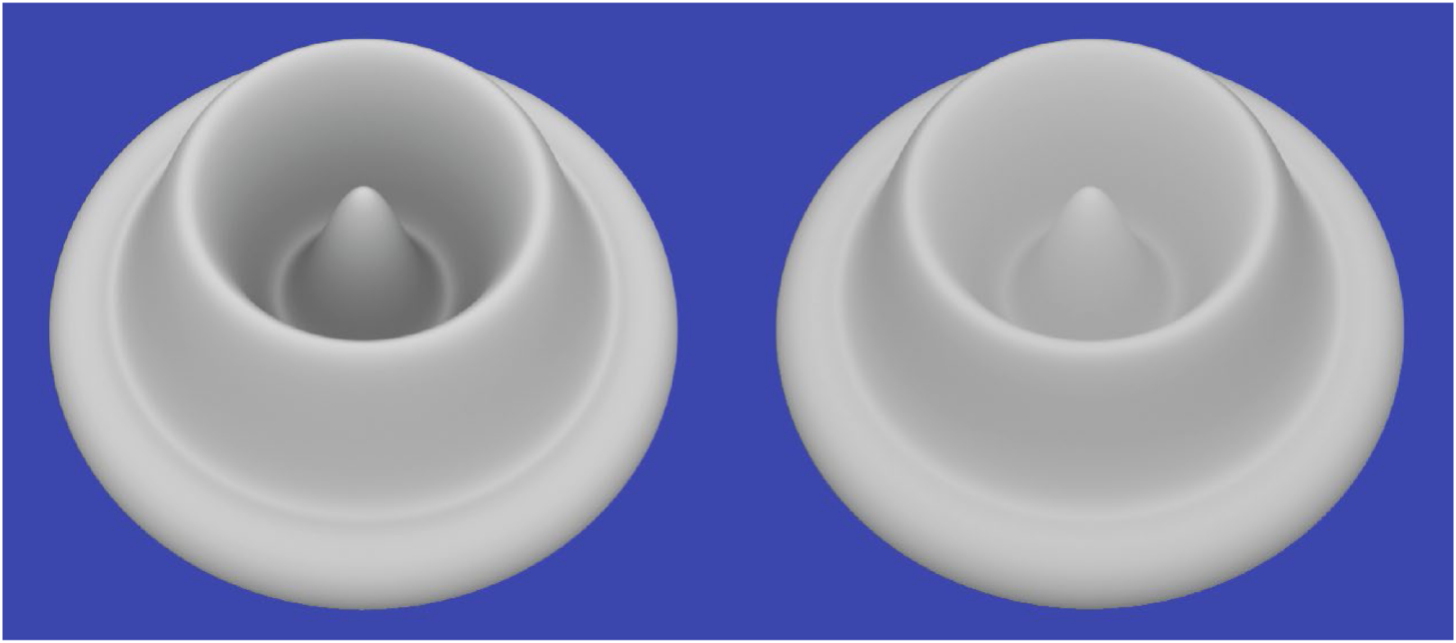
Effects of diffuse reflectance on indirect illumination. The surface on the left has a reflectance of .4, whereas the one on the right has a reflectance of .9. The inner concavity on the right appears brighter because the light can undergo a greater number of interreflections before its energy is dissipated by absorption.

The effects of surface interreflections are greatest in concave surface regions, because the light becomes trapped in those regions and illuminates the same surfaces on multiple bounces. This effect is demonstrated in Figure 25. Both images in this figure depict a radial cosine surface with diffuse overhead illumination as on a cloudy day. The surface depicted on the left has a reflectance of 0.4. Note that the inner part of the circular ridge appears darker than the outer part. This is because much of the sky is occluded in those regions so that they receive less illumination—an effect that is referred to as vignetting (Koenderink & Van Doorn, 2003; Langer & Zucker, 1994). The reflectance for the surface on the right has been increased to 0.9. This causes the light to undergo a greater number of bounces before all its energy is dissipated, and that increased illumination makes the internal part of the circular ridge appear brighter.

**Figure 26.**
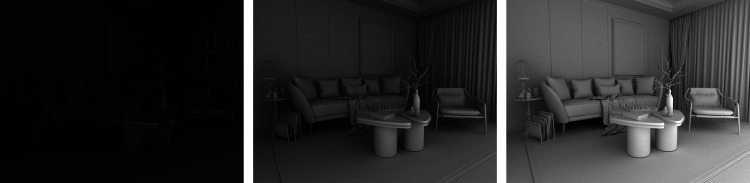
Three images of a white room. Moving from left to right, the images were rendered with 1, 5, and 15 indirect bounces, respectively.

Another convenient way to demonstrate the perceptual importance of surface interreflections is to examine scenes with no direct reflections at all. This is a fairly common occurrence for indoor environments. Most people find it unpleasant to look directly at luminous sources, so we typically hide them behind baffles or translucent diffusers. Consider the three images in Figure 26. They all depict a living room in which all the surfaces reflect 85% of the incident light. The scenes are all illuminated by a single spherical light source located behind a baffle so there is no direct illumination on any of the visible surfaces. These images were created with a renderer that makes it possible to manipulate the number of indirect bounces, and that is the only thing that differs between them. Moving from left to right, the images show the rendering results for 1, 5, and 15 indirect bounces, respectively.

### Specular Interreflections

**Figure 27.**
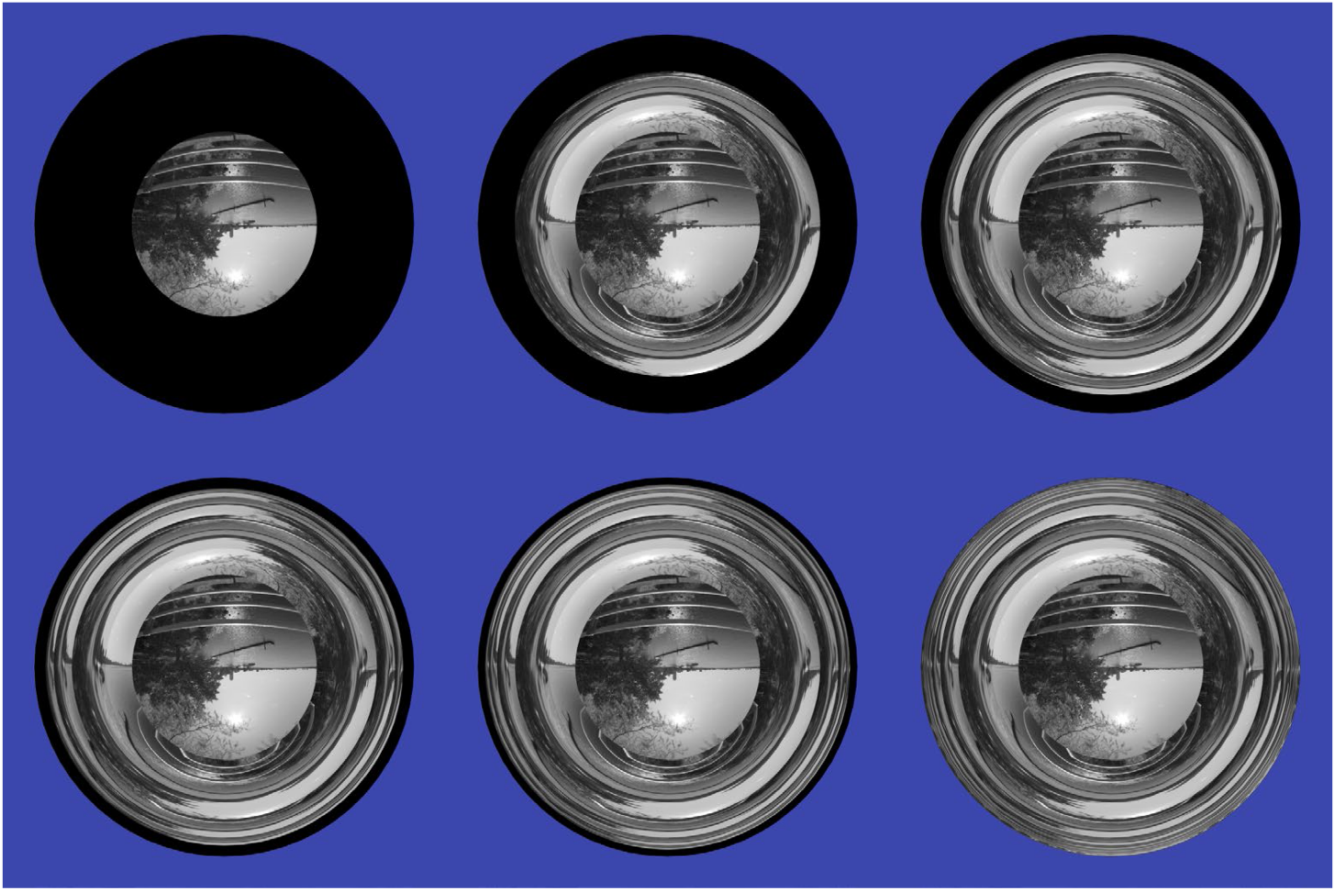
A concave hemispherical pit rendered with different numbers of reflective bounces. The top row from left to right shows 1, 2 and 3 bounces, respectively. The bottom row shows 4, 5, and 100 bounces.

The examples shown in Figures 25 and 26 all involve indirect illumination from diffuse reflections, but the behavior of specular interreflections can be even more interesting, as was demonstrated by Pont and Koenderink (2002, 2005). They performed a detailed analysis of surface interreflections inside the concavities of shiny materials. Consider the six images shown in Figure 27, which show a single hemispherical pit composed of a polished silver material with varying numbers of surface interreflections (see Todd & Norman, 2020). The image in the top left panel shows the visible structure that is produced by a single bounce of direct illumination. Note that the image of the surrounding scene is inverted, and that it is only visible in the central portion of the surface where light can be directly reflected toward the point of observation. The image in the top middle panel shows the visible structure that emerges after one additional indirect bounce. Note that this structure contains two circular bands: a large inner one where the surrounding scene appears upright, and a smaller outer one in which the scene appears inverted. As is shown in the remaining panels, each additional bounce produces two additional bands of visible structure that get progressively smaller as they approach the outer edge of the hemisphere.

**Figure 28.**
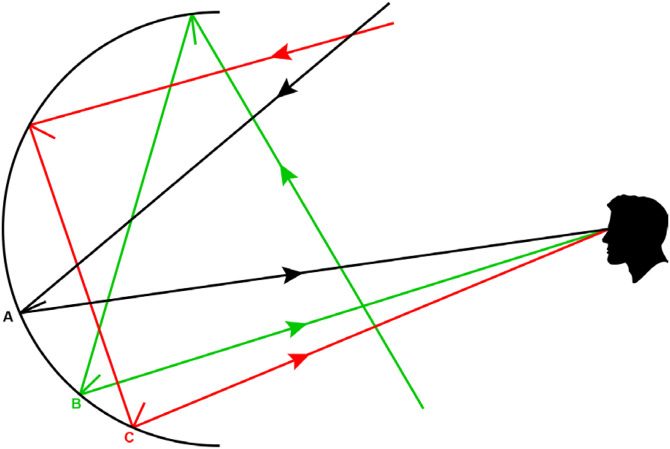
The light paths that reach the point of observation from three points on a concave hemispherical surface.

Although this banding behavior may appear at first blush to be counter-intuitive, it can easily be explained using geometrical optics. It is important to keep in mind that the simulated surface in Figure 27 is perfectly smooth. Thus, at any given point on the surface, there is only a single direction of illumination that will reflect light toward the point of observation, and that direction can be determined using backward ray tracing from the eye. Figure 28 shows three such points labeled A, B, and C on the lower half of a concave hemisphere, and the optical paths (colored black, green, and red) that would allow those points to reflect light toward the point of observation. Point A is in the region where direct reflections of the environment are visible, as shown by the path colored black. Note that the light that reaches the eye from that point comes from the upper part of the environment so that the image near A is inverted. Point B is located further out in the periphery. It can only reflect light toward the eye following an additional indirect bounce, as shown by the path colored green. Note how that path originates in the lower part of the environment so that the image near B is upright. Finally, point C is located even farther out in the periphery, and it also requires an additional indirect bounce to reflect light toward the eye as shown by the path colored red. However, note in that case that the path originates in the upper part of the environment, so that the image near C is again inverted. By moving farther and farther into the periphery, the number of required bounces to reflect light toward the eye increases, and the alternating bands of upright and inverted images repeat with higher and higher frequency.

**Figure 29.**
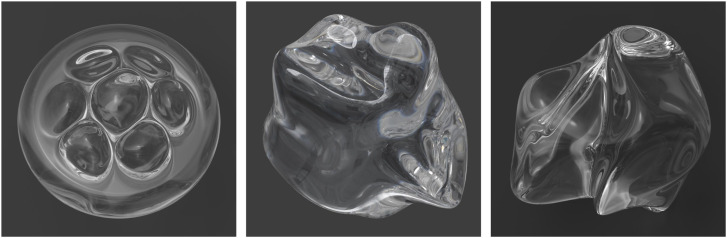
Three solid glass objects. The patterns of light and dark contours that surround the bumps on these surfaces are caused by multiple reflections within concave regions inside the glass material.

A similar pattern of optical banding can also occur inside transparent materials such as glass. When light hits a boundary from air to glass, almost all of its energy is transmitted except at high incident angles. Conversely, when light hits a boundary from glass to air at any incident angle above 41°, 100% of its energy will be reflected—a phenomenon that is referred to as total internal reflection. As a result of this high level of internal reflectance, glass materials can exhibit the same type of visible banding structure as is demonstrated in Figure 27 for shiny metals (see Todd & Norman, 2019, 2020). It is important to keep in mind, however, that this behavior occurs internally within glass objects such that all signs of curvature on its surface are reversed relative to how they appear from the outside. In other words, what appear to be ridges and bumps from the outside are actually valleys and pits from the inside. Figure 29 shows three glass objects with relatively complex patterns of concavity and convexity. Note in particular how alternating light and dark bands seem to surround all of the external bumps. These are the results of internal specular interreflections.

### Caustics

**Figure 30.**
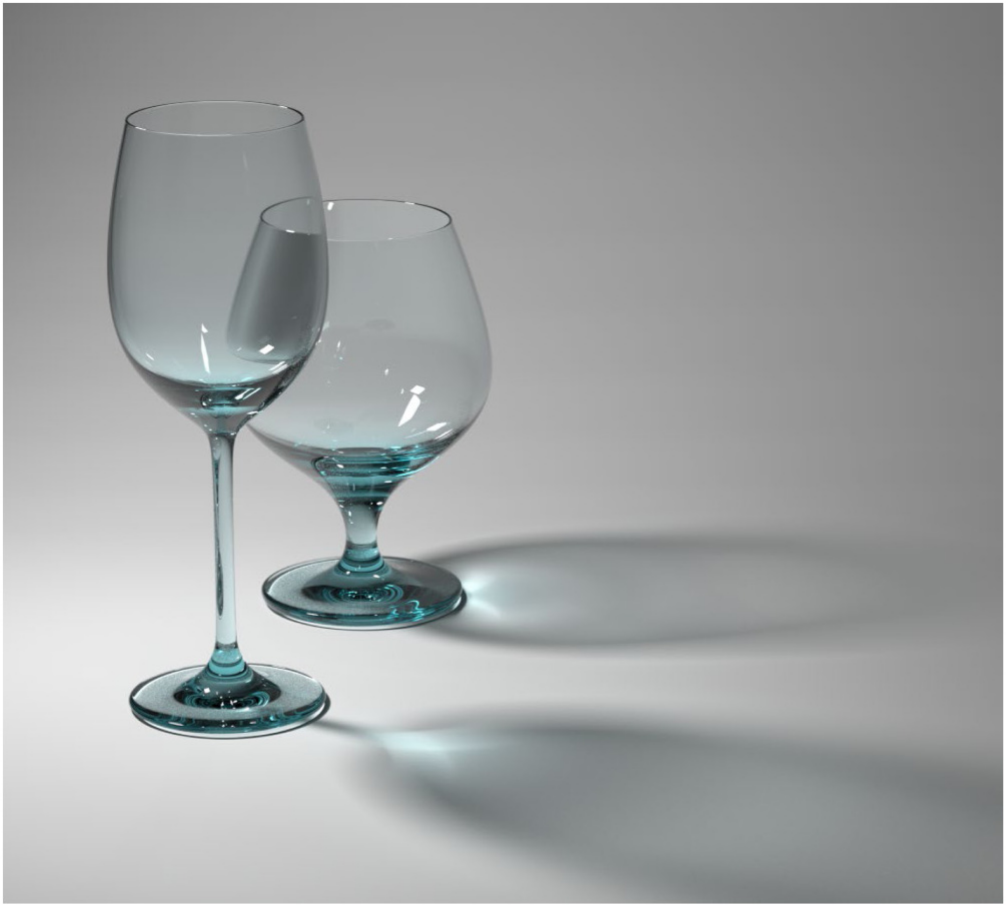
Two drinking glasses illuminated from the left. The bright regions within the shadows are called caustics.

The light that escapes from a transparent material can also illuminate other objects. For example, consider the image of two drinking glasses shown in Figure 30. Note that each glass casts a shadow that is darker along the edges than in the center. There is also a curved region of bright light called a caustic at the base of each shadow. The term caustic is derived from the Greek and Latin words for burnt or burning, and its usage in the context of optics refers to the fact that a concentration of light, especially sunlight, can cause combustible materials to burn. Caustics are defined as the envelope of light rays that have been reflected or refracted from a curved surface and projected onto a different surface. In the case of drinking glasses, the envelope of refracted light typically has a curved shape with a cusp.

### Psychological Research on Indirect Illumination

The first demonstration that indirect illumination can have a significant influence on human perception was presented by Gilchrist and Jacobson (1984) for the perception of lightness. Bloj et al. (1999) showed that indirect illumination can facilitate color constancy, and Madison et al. (2001) showed that it can also provide information about whether an object is in contact with the ground. More recent studies by Todd and Norman (2019, 2020) have investigated how internal specular interreflections can influence the perception of glass.

## Section 7: The Structure of Ambient Light

As a consequence of light scattering and surface interreflections, the spaces between objects in a scene become filled with light propagating in all directions. The first researcher to investigate the structure of this ambient light was the Russian physicist Andrey Gershun (1939), who published a monograph entitled “The light field,” which was later translated into English and published in the US in 1939. He assumed that the radiant energy of light is continuous in time and in space and that the flux of this energy varies continuously from point to point. This made it possible to describe the volumetric pattern of flux as a three-dimensional vector field (see also Kartashova et al., 2016; Moon & Spencer, 1981).

A similar insight about ambient light was arrived at independently by Gibson (1966). Gibson's ideas were primarily intuitive. He had no way to visualize the structure of ambient light, and he made no attempt to describe it mathematically. However, he was the first to recognize its importance for human perception. Gibson referred to this structure as the ambient optic array and he considered it to be a wellspring of potential information that observers can sample and manipulate to explore the environment.

One important difference between Gershon and Gibson is that Gershon determined the luminous flux at each point in space by averaging over all possible directions to obtain a single vector. Gibson would likely have disapproved of this approach. When he talked about sampling the optic array, he imagined an observer at a particular point in space who would be stimulated by the light converging on that point from all possible directions. It is the variation of light energy across different directions, and the variation that occurs across different vantage points that provides useful information about the structure of the environment.

### Interactions between Light Fields and Materials

**Figure 31.**
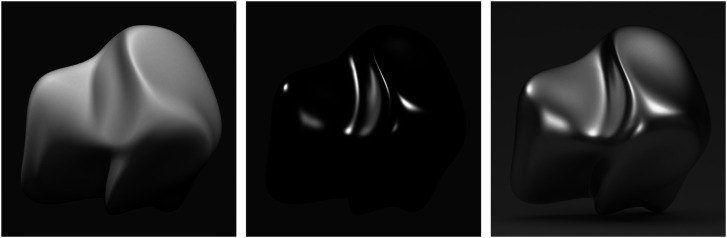
Images of three objects that are all illuminated by three small light sources in different directions. The one on the left has a Lambertian reflectance function and the other two both have chromium reflectance functions. For the scene on the right, the lights are located outside of a translucent box that contains the depicted object.

The light field is not just an abstract concept that we can take for granted in the study of ecological optics. It can have a profound impact on the perception of 3D shape and surface materials. In the earliest studies on shape from shading in the 1980s, the simulated objects were illuminated by a small number of point light sources. This approach works fine for surfaces with diffuse reflectance, but it does not produce acceptable results for purely specular surfaces like polished metal or glass. For example, the surface depicted in the left panel of Figure 31 has a Lambertian reflectance function and it is illuminated by three small light sources. Note that it is easily identified as a matte material and that the shape of the object is clearly specified by the pattern of image shading. The middle panel of Figure 31 shows the same surface with a chromium reflectance function. The shading in that case does not contain sufficient information that identify the complete shape of the surface, and it appears to be a shiny black dielectric material rather than a metal.

Studio photographers avoid this problem when dealing with metal materials by carefully controlling the pattern of illumination, so that a surface receives light from many different directions without introducing intense highlights from direct luminous sources (Hunter et al., 2007). One technique for achieving this is called *soft box* or *white box* photography, in which an object is photographed inside a translucent white box or tent (see Zhang et al., 2015). This allows soft light to enter from the outside, and the surface interreflections inside the box provide a richly structured pattern of ambient light. The image shown in the right panel of Figure 31 was created using a simulation of that method. The same three lights were employed as in the left and middle panels, but they were located outside of a translucent box that contained the depicted object (see Todd & Norman, 2018). Note how the appearance of metal is much more compelling than in the middle panel even though both objects have exactly the same reflectance function.

**Figure 32.**
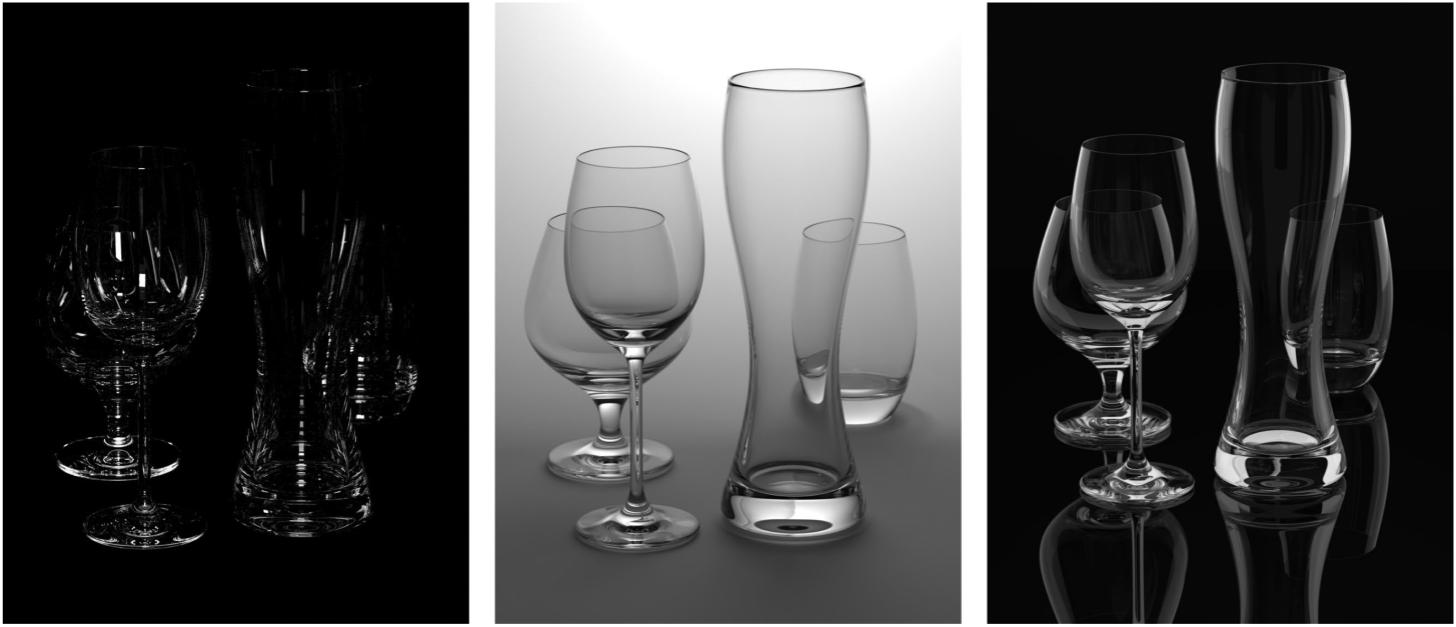
(Left) Four drinking glasses illuminated by a single light located slightly above the viewing direction. (Middle) The same four objects that are illuminated indirectly from reflections off the background surface. (Right) In this case, the objects are illuminated by two large luminous panels that are located to the left and right.

Glass is another material for which control of the light field is important to produce perceptually compelling images. Consider the image shown in the left panel of Figure 32 that depicts four drinking glasses against a black background illuminated by a rectangular area light positioned just above the point of observation. This image is a disaster from a photographic perspective, primarily because there is little or no light along the boundary of the objects that is reflected or transmitted toward the point of observation. As a result of that problem, the contours of the objects are mostly invisible.

To avoid problems with edge definition, photographers have worked out two classic illumination strategies that are often referred to as the bright-field method and the dark-field method. To better understand the logic of these approaches, it is important to recognize that there is only a narrow range of illumination directions for which regions along an object's boundary will reflect any light toward the point of observation. The secret to good edge definition is to control the light along those critical directions. In the bright-field method, there is no illumination in the critical directions so that the edges are dark, and the object is presented against a white background so that everything else is light. An example of this is shown in the middle panel of Figure 32. This scene is also illuminated by a single light, but that light is located behind the glasses so that it only provides direct illumination to the background surface. The glasses are then illuminated from behind by the reflections off that surface.

The dark-field method is exactly the opposite: The illumination is focused in the critical directions so that the edges are bright, and the object is presented against a black background so that everything else is dark. An example of that is shown in the right panel of Figure 32. The objects are illuminated by two large luminous panels that are located to the left and right. They are also resting on a highly reflective surface, which produces mirror reflections.

### Psychological Research on the Structure of Ambient Light

There have been a few psychophysical investigations in which the observers have been asked to make judgments about the light field by adjusting how a spherical probe would be shaded at different locations in visual space (Kartashova et al., 2016; Koenderink et al., 2007). There have also been many studies of the effects of lighting on the perception of material properties (e.g., Norman et al., 2020; Zhang et al., 2015) and on the perception of 3D shape from shading (e.g., Egan & Todd, 2015; Todd et al., 2023).

## Section 8: Practical Considerations for Psychological Research

The discussion thus far has focused primarily on the basic physical principles by which light interacts with surfaces in the natural environment. One of the goals of perceptual psychology is to identify the possible sources of information by which observers are able to identify the shapes and material properties of visible objects from patterns of visual stimulation, and a typical way of achieving that is to analyze the stimulus differences that are diagnostic about specific perceptual distinctions like matte versus shiny or opaque versus transparent.

It is important to point out, however, that some physical differences among patterns of stimulation may not be perceptually relevant. Consider the case of chromatic dispersion as shown in Figure 15. It is quite likely that experts on the production of glass might be able to judge the lead content of a glass material from the extent of chromatic dispersion. Although these effects are clearly noticeable to naive observers, they may not be perceptually meaningful without explicit instructions. It is probably not surprising, therefore, that many renderers do not implement that effect because it is computationally costly and has a minimal impact on human perception. This is also true for thin film interference. The specific colors produced by that effect allow physicists to determine the thickness of thin films with a high degree of accuracy, but those distinctions are likely to be meaningless for naive observers.

The Fresnel effect is another phenomenon that cannot be simulated on some renderers and can only be approximated in others. It remains to be determined if these effects are perceptually relevant for distinguishing between metals and dielectric materials. If both types of materials are present in the same scene with the same illumination, the luminance reflected off metal objects is many times greater than the visible reflections off shiny black plastic. It is possible to control for that by presenting each material with a different illumination, so that their mean luminance is equated. In principle, there is still potential information for distinguishing metals from dielectrics based on the distribution of image intensities between regions near the center of an object and those that are closer to its occlusion boundary, as shown in Figure 21. Readers can judge for themselves whether that information is sufficient to reliably distinguish metal and shiny black materials (see Todd & Norman, 2018). Another aspect of the Fresnel effect that is almost certain to be perceptually relevant is the difference in color between metals and dielectric materials, as shown in Figure 24. This is a particularly interesting issue for future research.

**Figure 33.**
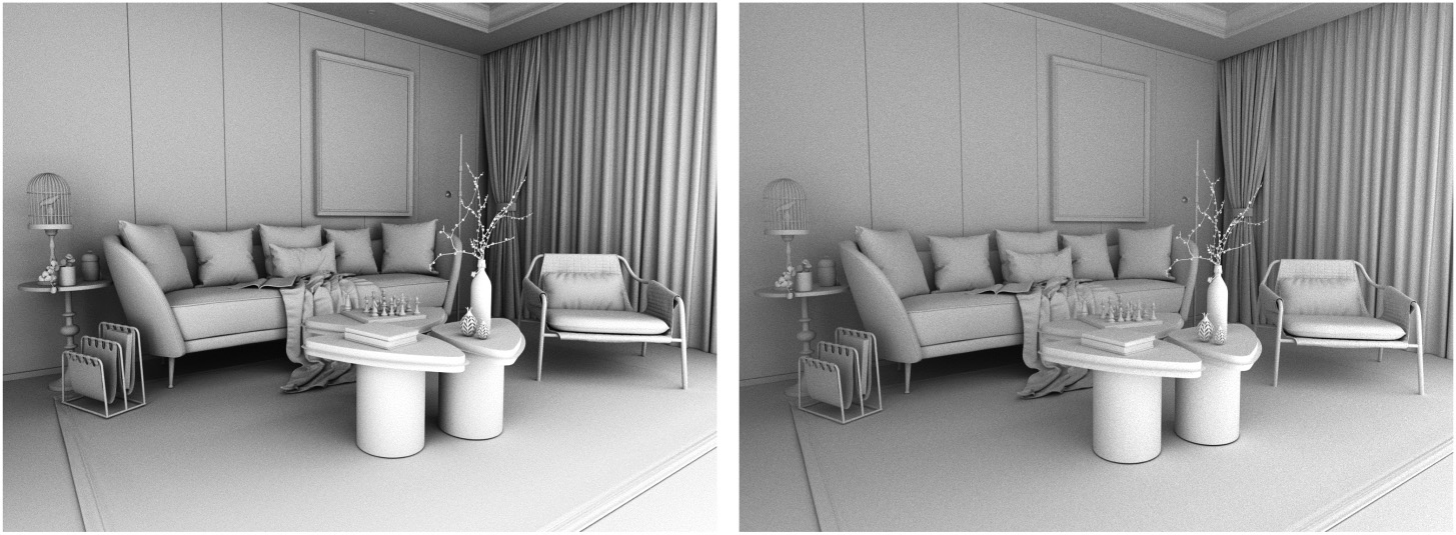
A graphic simulation of a living room with three indirect bounces (left) and ten indirect bounces (right). Both images have been adjusted so that they have the same mean luminance, but the one on the left has a higher variance.

It has long been known that indirect illumination can have a significant influence on human perception. This was first demonstrated almost 40 years ago by Gilchrist and Jacobson (1984) using a display similar to Figure 25 that they created with real objects. Most modern renderers allow the user to control the number of indirect bounces in a simulation, and there is an old rule of thumb among researchers in perception that two or three bounces are sufficient to capture the relevant information. The validity of that rule depends on the complexity of the depicted scene. If a globally convex object is presented in empty space, then indirect illumination has no effect at all on its pattern of image shading. The effects of indirect reflective bounces only become visible when there are surface concavities where light can become trapped. This is especially true for enclosed spaces like the one depicted in Figure 33. The image on the left of that figure was simulated with three indirect bounces, whereas the one on the right was simulated with ten. Both images have been adjusted to have the same mean luminance, but the one on the left has a noticeably higher variance (see also Gilchrist & Jacobson, 1984).

Indirect bounces can be particularly important for images of specular materials. This is demonstrated quite clearly in Figure 27, which shows how multiple bounces are necessary to fill out the shading within concavities on metal surfaces. This is also true for glass materials to produce the banding structures shown in Figure 29. The V-Ray renderer has an interesting feature that assigns a color to mark the amount of undissipated energy at each pixel that remains after the last bounce of a graphics simulation. Todd and Norman (2019) exploited that feature to investigate the dissipation of energy within solid and hollow glass objects like the ones shown in Figure 29. Their results showed that solid glass objects can require up to 20 indirect bounces for all the energy to dissipate, and that hollow glass objects can require up to 100 bounces. Hollow objects produce a larger number of internal bounces than solid ones because light becomes trapped inside the glass shell so that most of the rays hit the boundaries at relatively high incident angles that are within the range of total internal reflection. This is the same principle by which fiber optic cables are able to transmit light over long distances.

## Conclusions

This article has provided an overview of how the concept of light has evolved over the course of human history. It has described how light interacts with surfaces in the natural environment, and it has examined how those interactions modulate the patterns of visual stimulation for human observers. It is important to keep in mind that there is large literature on the perception of 3D shape and surface materials from patterns of image shading, and the present discussion has made no attempt to summarize that research other than to highlight a few relevant references. Rather, the goal of this article is to provide the necessary background information to help students and young researchers more easily understand that literature, and to better appreciate its limitations.

## Supplemental Material

**Figure:** Video 1.

sj-gif-1-ipe-10.1177_20416695241279929 - Supplemental material for A tutorial on the physics of light and image shading Supplemental material, sj-gif-1-ipe-10.1177_20416695241279929 for A tutorial on the physics of light and image shading by James T. Todd in i-Perception
