# Supplementary figures and images for: A tutorial on the physics of light and image shading
Source: Iperception. 2024 Sep 30;15(5):20416695241279929. doi: 10.1177/20416695241279929 (PMC11483731; doi:10.1177/20416695241279929)

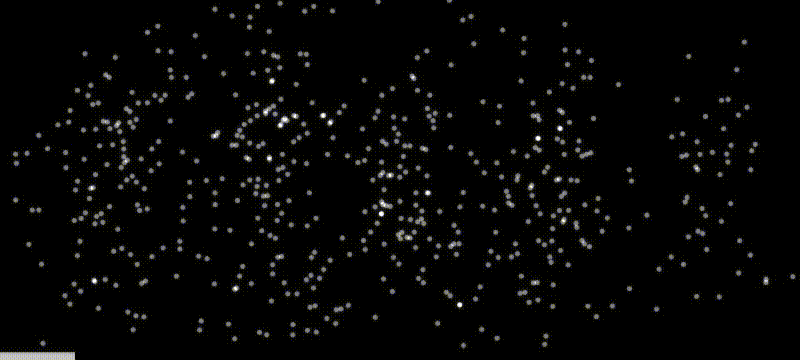

Supplement: sj-gif-1-ipe-10.1177_20416695241279929 - Supplemental material for A tutorial on the physics of light and image shading [file sj-gif-1-ipe-10.1177_20416695241279929.gif]
